# Universal genotyping reveals province-level differences in the molecular epidemiology of tuberculosis

**DOI:** 10.1371/journal.pone.0214870

**Published:** 2019-04-03

**Authors:** Jennifer L. Guthrie, Alex Marchand-Austin, Kirby Cronin, Karen Lam, Daria Pyskir, Clare Kong, Danielle Jorgensen, Mabel Rodrigues, David Roth, Patrick Tang, Victoria J. Cook, James Johnston, Frances B. Jamieson, Jennifer L. Gardy

**Affiliations:** 1 School of Population and Public Health, University of British Columbia, Vancouver, Canada; 2 Public Health Ontario, Toronto, Canada; 3 National Microbiology Laboratory, Public Health Agency of Canada, Winnipeg, Canada; 4 British Columbia Centre for Disease Control, Public Health Laboratory, Vancouver, Canada; 5 British Columbia Centre for Disease Control, Vancouver, Canada; 6 Department of Pathology and Laboratory Medicine, University of British Columbia, Vancouver, Canada; 7 Respiratory Medicine, University of British Columbia, Vancouver, Canada; 8 Department of Laboratory Medicine and Pathobiology, University of Toronto, Toronto, Canada; St Petersburg Pasteur Institute, RUSSIAN FEDERATION

## Abstract

**Objectives:**

Compare the molecular epidemiology of tuberculosis (TB) between two large Canadian provinces–Ontario and British Columbia (BC)–to identify genotypic clusters within and across both provinces, allowing for an improved understanding of genotype data and providing context to more accurately identify clusters representing local transmission.

**Design:**

We compared 24-locus Mycobacterial Interspersed Repetitive Units-Variable Number of Tandem Repeats (MIRU-VNTR) genotyping for 3,314 Ontario and 1,602 BC clinical *Mycobacterium tuberculosis* isolates collected from 2008 through 2014. Laboratory data for each isolate was linked to case-level records to obtain clinical and demographic data.

**Results:**

The demographic characteristics of persons with TB varied between provinces, most notably in the proportion of persons born outside Canada, which was reflected in the large number of unique genotypes (*n* = 3,461). The proportion of clustered isolates was significantly higher in BC. Substantial clustering amongst non-Lineage 4 TB strains was observed within and across the provinces. Only two large clusters (≥10 cases/cluster) representing within province transmission had interprovincial genotype matches.

**Conclusion:**

We recommend expanding analysis of shared genotypes to include neighbouring jurisdictions, and implementing whole genome sequencing to improve identification of TB transmission, recognize outbreaks, and monitor changing trends in TB epidemiology.

## Introduction

Tuberculosis (TB) remains a major public health issue in Canada. Molecular techniques, such as 24-locus Mycobacterial Interspersed Repetitive Units–Variable Number Tandem Repeat (MIRU-VNTR) genotyping, have improved understanding of TB epidemiology, and many jurisdictions are adopting routine genotyping of all *Mycobacterium tuberculosis* (*Mtb)* isolates [1–3]. Within Canada, the province of Ontario–which has the largest number of TB cases of any Canadian province [4]–was an early adopter of universal genotyping, using MIRU-VNTR to genotype the first culture-positive isolate for each case since mid-2007 [5]. Interpreting these data in the context of linked clinical and demographic information has facilitated both contact tracing and outbreak detection in the province [6]. More recently, British Columbia (BC) retrospectively genotyped all first culture-positive isolates since 2005 [1], and implemented universal genotyping in 2015.

TB incidence rates in Ontario and BC are nearly identical, with 4.5 and 4.6 cases per 100,000 population respectively [7]. Together, these two provinces represent a substantial burden of disease in Canada, accounting for >50% of the nation’s TB cases, with rates in both settings largely driven by reactivation of latent TB infection (LTBI) in persons born outside Canada [4]. Both provinces are popular destinations for immigrants, with the multi-cultural cities of Toronto and Vancouver attracting many newcomers [8]. Vancouver has a high proportion of immigrants from Asia whereas Toronto is more diverse and, in addition to people from Asia, also has many immigrants from Africa, the Caribbean, and Latin America [8]. Furthermore, despite the distance between these provinces, there is substantial interprovincial travel and migration, with ~15,000 individuals reportedly migrating from Ontario to BC and vice-versa in 2016/17 [9]. Migrants of all types frequently lack support networks and are at greater risk for homelessness and other factors associated with increased risk of TB reactivation or infection [10]. This is particularly true in BC, where under-housed migrants from other provinces–some of whom are experiencing mental illness, addictions, and/or chronic health conditions [10,11]–are thought to be attracted to Vancouver by the temperate climate.

Each Canadian province/territory works independently towards TB prevention and care and contributes data towards national TB surveillance programs; however, there is currently no national-level TB molecular surveillance program. Although not unique to Canada, cross-jurisdictional issues, including funding, privacy, platforms for disseminating information, and necessary support personnel, as well as questions surrounding the benefits that such a program would offer, have prevented implementation thus far. Having completed universal MIRU-VNTR genotyping dating back over a decade, Ontario and BC have the most extensive collections of MIRU-VNTR genotyped isolates in Canada. This provides a unique opportunity to compare the molecular epidemiology of TB between these large immigrant-receiving provinces and demonstrate the added value of genotype data shared between different jurisdictions by providing context to the genotypes observed within each region. The resulting insights are key to understanding genotypic clustering as it relates to local spread of TB, and establishing proof of concept for a national genotyping program.

## Methods

### Study setting and design

Ontario and BC are the first and third most populous Canadian provinces, respectively, with 14.2 and 4.8 million inhabitants [9], and rank first and second for the highest population proportion of immigrants, at 28.5% for Ontario and 27.6% for BC [12]. All *Mycobacterium tuberculosis* (*Mtb*) isolates are either identified in culture at the provincial reference laboratories–Public Health Ontario Laboratory (PHOL) and British Columbia Centre for Disease Control Public Health Laboratory (BCPHL), or submitted for reference testing from other laboratories. The study population included all culture-positive TB cases residing in Ontario or BC at TB treatment initiation, with a first *Mtb* sensu stricto isolate received from 2008 through 2014. Therefore, 3,314 Ontario and 1,602 BC isolates, representing 75.2% and 79.7% of all notified TB diagnoses during this time period in the respective provinces were included. For individuals with a reoccurrence during the study period indicative of relapse–successful completion of treatment and identical MIRU-VNTR results for both episodes (Ontario: *n* = 5, BC: *n* = 9)–only data from the first episode was included.

Ethics approval was granted by Public Health Ontario (#2016–058.0), and the University of British Columbia (certificate #H12-00910).

### Diagnosis and case information

All provinces/territories follow the Canadian Tuberculosis Standards [4] for investigation, management and reporting of active TB. Case-level clinical and demographic data, including age, sex, birthplace, and disease site were extracted from each province’s independently held reportable disease registry–the integrated Provincial Health Information System (iPHIS) in Ontario and Panorama in BC–and were linked to the genotype results in their respective provinces. To assess genotyping in the context of urban/rural regions, community type was determined using Statistics Canada-defined health region Peer Groups (A–I) to effectively compare health regions with similar characteristics across provinces/territories [13]. We grouped these into four higher-level categories: Metro (G), Urban, high-density (A, H), Urban, moderate-density (B), Rural/Remote (C–E, I). A description of each peer group is provided by Statistic Canada [13].

### Genotyping by 24-locus MIRU-VNTR

Using standard methods [14], we successfully MIRU-VNTR genotyped 97.8% (3,314/3,388) of Ontario isolates and 99.8% (1,602/1,605) of BC. Isolates lacking an amplicon peak at any locus had MIRU-VNTR repeated with newly extracted DNA, and where there remained no peak at a single locus–excluding loci 2163 and 2165, which are known to be absent in some strains [15]–the locus was coded as missing data and the isolate included in the analyses. Major lineage (L) was predicted using the TB-Insight webtool [16], and categorized as Indo-Oceanic (L1), East-Asian (L2), East-African-Indian (L3) or Euro-American (L4). Sub-lineage was determined using TBminer [17] for isolates in which the major lineage predicted was concordant with TB-Insight. In cases where the major lineage was discordant between these prediction tools the TB-Insight lineage was used. We defined an intraprovincial cluster as ≥2 isolates with an identical MIRU-VNTR pattern within a province, and an interprovincial cluster as one or more isolates sharing an identical genotype across the two provinces. Genotypic clusters within each major lineage were visualized using a Minimum Spanning Tree (MST) created in PHYLOViZ 2.0 [18] and coloured by province. To graphically represent the relationship between the number of isolates contributing to a genotype match between the provinces, we displayed interprovincial clusters using a circular chord diagram according to the number of isolates contributing to an interprovincial genotype cluster: single (1 isolate), small (2–9 isolates), large (≥10 isolates).

### Statistical analysis

We compared case-level characteristics using a Chi-square test for categorical variables (Fisher’s Exact test where appropriate), and a t-test for continuous variables. Intraprovincial clustering proportions were compared using Chi-square. To calculate the clustered proportion potentially attributable to local transmission, we used the “*n*−1” method in which the first case of each cluster is assumed to have initiated the cluster and is subtracted from the total number of clustered isolates [19]. We used logistic regression to examine factors associated with interprovincial clustering, calculating the odds ratio (OR), adjusted OR (aOR), and 95% confidence interval (CI). A complete-case analysis strategy (excluded records with missing data: *n* = 109 [2.2%]) was used, with stepwise backward selection of variables following Akaike Information Criterion minimization. All statistical analyses were conducted using R statistical software (v3.4.1).

## Results

### Descriptive epidemiology

The study population included a total of 4,916 cases (3,314 in Ontario and 1,602 in BC) with a diagnosis of culture-positive TB from 2008–2014. The median age was 46 in Ontario with an interquartile range (IQR) of 30–67 –significantly lower than in BC (53 years, IQR: 37–72), *p*<0.001. Case distribution by community type varied between the provinces (Table 1), with many Ontario cases residing in Metro areas (47.0%) and most BC cases in high-density urban areas (55.7%). Notably, a higher proportion of BC cases resided in rural/remote regions (11.8% versus 4.1%). Country of birth was available for 97.5% of individuals, the majority of whom were born outside Canada (Table 1); however, the proportion varied significantly between Ontario (91.3%) and BC (73.5%). Furthermore, Ontario had a higher proportion of recent immigrants–those arriving within the last five years–(*n* = 1,024; 35.5%) compared to BC (*n* = 309; 27.8%). BC had a higher proportion of persons with respiratory disease (85.1%) versus Ontario (74.9%).

**Table 1:** Demographic and clinical characteristics of culture-positive cases 2008–2014, Ontario (*n* = 3,314) and British Columbia (*n* = 1,602).

Characteristic	Ontario	British Columbia	p-value†
	*n* (%)*	*n* (%)*	
Age, years			<0.001
0–14	51 (1.5)	20 (1.2)	
15–34	1001 (30.2)	339 (21.2)	
35–54	952 (28.7)	470 (29.3)	
55–74	747 (22.5)	425 (26.5)	
75+	563 (17.0)	348 (21.7)	
Sex‡			
Male	1838 (55.5)	939 (58.6)	0.041
Community type			<0.001
Metro	1556 (47.0)	449 (28.0)	
Urban, high-density	1132 (34.2)	893 (55.7)	
Urban, moderate-density	490 (14.8)	71 (4.4)	
Rural/Remote	136 (4.1)	189 (11.8)	
Birthplace§			<0.001
Canada	284 (8.7)	412 (26.5)	
Foreign-born continent||			<0.001
Asia	2282 (77.2)	1017 (89.0)	
Africa	382 (12.9)	50 (4.4)	
Europe	167 (5.6)	45 (3.9)	
Americas	120 (4.1)	24 (2.1)	
Oceania	5 (0.2)	7 (0.6)	
Time in Canada#			<0.001
< 5 years	1024 (35.5)	309 (27.8)	
≥ 5 years	1860 (64.5)	801 (72.2)	
Disease Site			<0.001
Respiratory	2256 (68.1)	1250 (78.0)	
Non-Respiratory	832 (25.1)	238 (14.9)	
Respiratory + Non-Respiratory	226 (6.8)	114 (7.1)	

*Percentages have been rounded and my not total 100%.

†Chi-square tests.

Data unavailable:

‡ON: n = 4;

§ON: n = 60, BC: n = 45;

||ON: n = 14, BC: n = 2;

#ON: n = 86, BC: n = 35.

### TB isolates in BC are more likely to be clustered by MIRU-VNTR

To understand the patterns of clustering, we examined the number and size distribution of genotypic clusters. MIRU-VNTR genotyping grouped the Ontario *Mtb* isolates into 290 clusters, with a mean cluster size of four isolates (size range: 2–49), yielding a clustered proportion of 31.8% (S1 Table). In BC, we identified 134 clusters, with an average cluster size of five isolates (size range: 2–68) and an overall clustered proportion of 40.5%–significantly higher than found in Ontario (*p*<0.001). Using the *“n*−1” method, [19] the number of infections potentially attributable to local transmission was 1,053 (23.0%) in Ontario and 649 (32.1%) in BC, indicating that while the overall number of cases in BC is lower, the proportion of TB diagnoses that may be the result of local transmission is higher.

In both provinces, more than half the clusters– 56.7% in Ontario and 54.9% in BC–contained only two individuals, likely representing single transmission events for which there is little opportunity for intervention. In contrast, large clusters may represent ongoing transmission where there is more opportunity for preventive measures. Only a few large clusters of ≥10 individuals were present in either province (Ontario: *n* = 11 [3.8%], BC: *n* = 10 [7.5%]). Differences in the clustered proportion between the two provinces was largely driven by clustering amongst Canadian-born persons (Ontario: *n* = 142 [50.0%], BC: *n* = 312 [75.7%]), as the clustered proportion was similar for persons born outside Canada (Ontario: *n* = 892 [30.0%], BC: *n* = 322 [28.1%]), a finding that suggests BC experiences more local TB transmission.

### Interprovincial clustering occurs frequently between Ontario and BC

**Fig 1:**
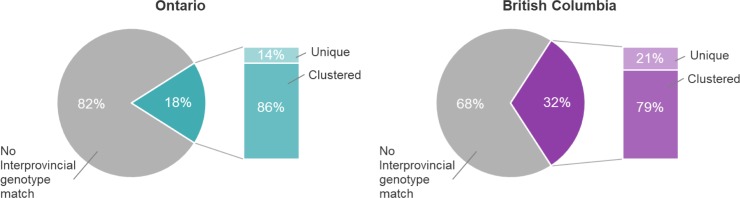
Intra- and interprovincial 24-locus MIRU-VNTR genotypic clustering, Ontario and British Columbia (2008–2014). Each pie represents the proportion of isolates within the province that have a genotype match in the other. For the group that does have an interprovincial match, the stacked bar graphs show the relative frequency of isolates that are clustered or unique within the province.

In total, we observed 3,461 distinct MIRU-VNTR patterns (S2 Table) across both provinces. Although only 175 of these patterns were detected in both Ontario and BC (S1 Fig), this constituted 22.4% (1,102/4,916) of all study isolates sharing a genotype pattern across both provinces– 595 (18.0%) Ontario isolates and 507 (31.6%) BC isolates. To determine whether the isolates with MIRU-VNTR matches across the two provinces represented unique genotypes or common clusters within a province, we examined MIRU-VNTR clustering in each province. We found that the majority of these interprovincially matched isolates were also clustered within their respective provinces– 85.5% (509/595) in Ontario and 79.1% (401/507) in BC (Fig 1). The considerable number of interprovincial matches that also clustered with isolates within their respective provinces demonstrates that common genotypes occur frequently across geographically disparate regions.

**Fig 2:**
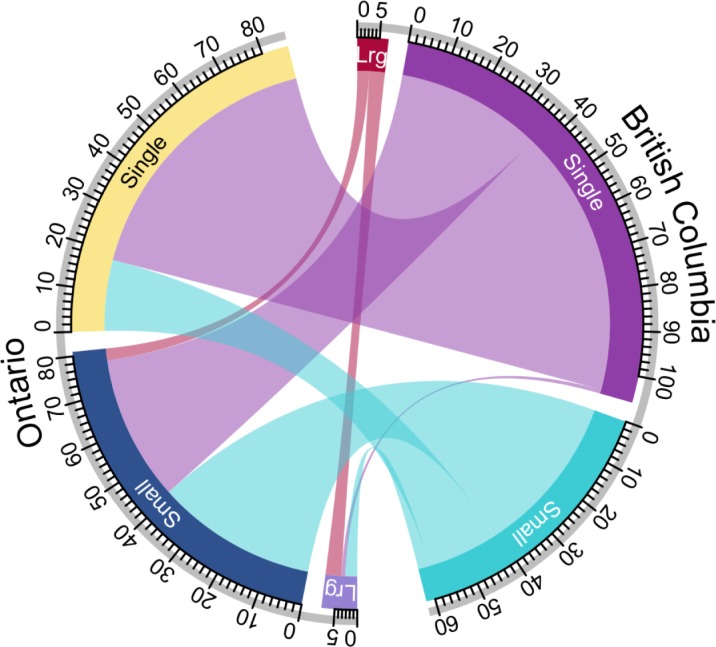
Interprovincial genotype matches by cluster size within a province. A circular chord diagram visualizing the number (indicated by tick marks) of interprovincial 24-locus MIRU-VNTR genotype matches between Ontario (left) and British Columbia (right) from 2008–2014, grouped by the number of isolates within each province sharing the matched genotype: single (1 isolate), small (2–9 isolates), large (≥10 isolates). Flow width indicates the number of genotypes. For example 20 single isolates each with a different MIRU-VNTR pattern in Ontario are genotype matches to 20 different small British Columbia MIRU-VNTR clusters.

We used multivariable logistic regression to investigate independent factors associated with interprovincial genotype matches (Table 2). We found increased odds of interprovincial matching for BC isolates (aOR 2.1, 95%CI: 1.8–2.5) compared to Ontario isolates–indicating Ontario had considerably more unique MIRU-VNTR patterns. Additionally, the odds of matching were higher for Canadian-born persons (aOR 2.5, 95%CI: 1.9–3.2), and those with a non-L4 *Mtb* isolate (aOR range: 1.9–4.7). Individuals residing in a Metro area had 1.8 times the odds of their isolate belonging to an interprovincial cluster (95%CI: 1.2–2.5) compared to those residing in a rural/remote region. From a public health perspective, understanding the discriminatory power of MIRU-VNTR for investigating potential transmission is key. Comparing genotypic matches across the provinces–particularly those representing intraprovincial clusters–can reveal whether these clusters represent a common genotype circulating in a specific region of the world or are instead the result of local transmission; the former scenario is more likely when the same genotype is also common in a distant province. To examine factors that could be related to cluster size and MIRU-VNTR matches between the provinces we restricted the sample to include only isolates contributing to an interprovincial genotypic cluster and compared single versus multiple contributors to a cluster. We observed very similar trends to the factors associated with overall interprovincial clustering (S3 Table). Furthermore, upon examination of cluster composition (Fig 2), we found 68 of the 175 interprovincial clusters were comprised solely of a single isolate detected in each province– 80.9% were L1, L2, or L3 clusters and 93.9% of these isolates were identified in persons born outside Canada (S2 Fig). This suggests that genotypic matching between the provinces is often the result of strains with MIRU-VNTR patterns common to the place of origin in persons born outside Canada and likely representing LTBI reactivation–key information for understanding clustering within provinces.

**Table 2:** Distribution, frequency, and logistic regression analysis of factors associated with interprovincial genotypic clustering of *Mycobacterium tuberculosis* isolates between Ontario and British Columbia 2008–2014 (*n* = 4,807).

	Interprovincial Genotype Match		Interprovincial Genotype Match	
	Yes	No	Yes vs. No	Yes vs. No
Characteristic	*n* (%)	*n* (%)	OR (95%CI)	aOR* (95%CI)
Total	1075 (22.4)	3732 (77.6)		
Age, years				
0–14	13 (18.3)	58 (81.7)	0.8 (0.4–1.4)	0.6 (0.3–1.1)
15–34	303 (22.8)	1026 (77.2)	Reference	Reference
35–54	357 (25.4)	1049 (74.6)	1.2 (1.0–1.4)	1.1 (0.9–1.3)
55–74	251 (22.0)	889 (78.0)	1.0 (0.8–1.2)	0.9 (0.7–1.1)
75+	151 (17.5)	710 (82.5)	0.7 (0.6–0.9)	0.6 (0.5–0.8)
Sex				
Female	465 (22.1)	1638 (77.9)	Reference	Reference
Male	610 (22.6)	2094 (77.4)	1.0 (0.9–1.2)	1.0 (0.9–1.2)
Province of residence				
Ontario	578 (17.8)	2672 (82.2)	Reference	Reference
British Columbia	497 (31.9)	1060 (68.1)	2.2 (1.9–2.5)	2.1 (1.8–2.5)
Community type				
Metro	458 (23.2)	1518 (76.8)	1.3 (1.0–1.8)	1.8 (1.2–2.5)
Urban, high-density	470 (23.6)	1520 (76.4)	1.3 (1.0–1.8)	1.6 (1.1–2.2)
Urban, moderate-density	92 (16.8)	455 (83.2)	0.9 (0.6–1.3)	1.4 (1.0–2.2)
Rural/Remote	55 (18.7)	239 (81.3)	Reference	Reference
Birthplace				
Canada	192 (27.6)	503 (72.4)	1.4 (1.2–1.7)	2.5 (1.9–3.2)
Outside Canada	883 (21.5)	3229 (78.5)	Reference	Reference
Lineage				
L1	346 (26.2)	973 (73.8)	2.1 (1.8–2.5)	3.1 (2.5–3.9)
L2	340 (35.1)	628 (64.9)	3.2 (2.7–3.9)	4.7 (3.7–5.8)
L3	156 (17.3)	744 (82.7)	1.2 (1.0–1.6)	1.9 (1.5–2.4)
L4	233 (14.4)	1387 (85.6)	Reference	Reference

Abbreviations: OR—odds ratio; CI—confidence interval.

*Adjusted for age, sex, province, community type, birthplace, lineage.

Differentiating strains by lineage and sub-lineage revealed dominant sub-lineages–L1_EA2, L2_Beijing, and L3_CAS which were associated with particular geographic regions (S3 Fig)–most notably, 93.3% of individuals born in Philippines had an L1_EA2 isolate. Two MIRU-VNTR patterns (ON253/BC011: 5224341442218A7263223363, ON267/BC021: 5224341442219A7263223363) within this sub-lineage were commonly seen in both Ontario and BC.

### *Mtb* population structure reveals large BC-based Lineage 4 clusters

**Fig 3:**
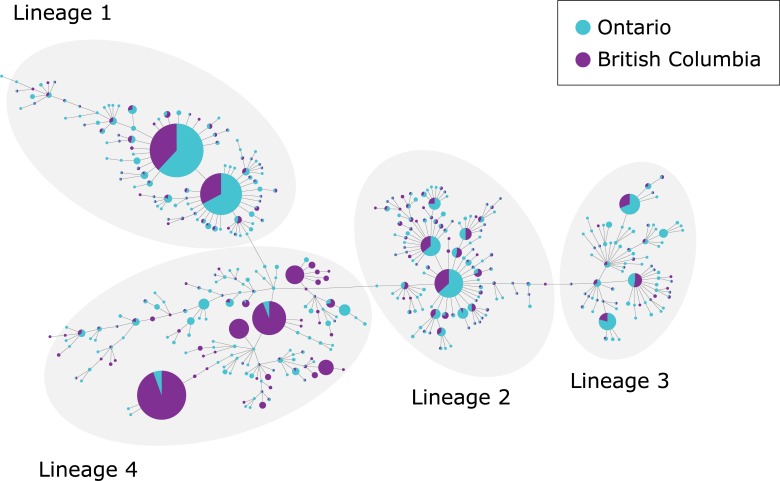
Minimum spanning tree analysis of 24-locus MIRU-VNTR of the 1,894 intra- and interprovincially clustered isolates with lineage indicated, Ontario and British Columbia (2008–2014). The size of each circle is proportional to the number of isolates. Classification of genotypes by province is visualized by colour coding.

We visualized the 1,894 isolates that were intra- and/or interprovincially clustered using a minimum spanning tree (Fig 3), revealing 17 large clusters (≥10 persons), many of which were observed in both Ontario and BC. Recognizing that MIRU-VNTR overestimates transmission in non-L4, [20] and that local transmission is more likely to occur amongst Canadian-born persons, we examined these clusters in the context of lineage and birthplace (Table 3). Clusters of non-L4 isolates were observed in persons born outside Canada, and all but one of these clusters spanned both provinces, suggesting that rather than local transmission, these clusters may reflect reactivation of strains acquired overseas. Clusters involving predominantly Canadian-born persons tended to occur exclusively within one province or the other and in different community types–Metro and high-density urban in Ontario, largely rural/remote in BC. However, seven isolates with genotypes matching two large BC outbreaks (BC002 and BC012)–one of which has been previously described [21]–appeared in Ontario. Cases in these two BC-based clusters arose throughout the study period, whereas those with matching MIRU-VNTR genotypes in Ontario were seen sporadically (S4 Fig), with the first case matching the BC002 genotype diagnosed in 2011, and the initial Ontario case matching BC012 diagnosed in 2008. The large number of BC cases diagnosed in 2008 suggests these two strains were present in BC prior to the study period and that the individuals in Ontario potentially acquired their infections through travel to BC or contact with an individual that had spent time in BC.

**Table 3:** Characteristics of 24-locus MIRU–VNTR large clusters (≥10 individuals) by predominant birthplace, median age (years), sex ratio, community type, and lineage: Ontario and British Columbia, 2008–2014.

	Ontario					British Columbia					
Cluster ID	Cluster Size	Birthplace* (%)	Median Age (IQR)	Sex M:F	Community Type† (%)	Cluster Size	Birthplace* (%)	Median Age (IQR)	Sex M:F	Community Type† (%)	Lineage
Interprovincial Clusters											
Canadian-born											
ON059/BC002	4	Canada (100)	30 (29–39)	NA‡	uMD/R (75)	68	Canada (88)	51 (43–57)	12.6	uHD (68)	L4_X
ON065/BC012	3	Canada (50)	51 (50–68)	2.0	uMD/R (67)	46	Canada (91)	49 (41–56)	2.5	uHD (52)	L4_H3
Non-Canadian-born											
ON253/BC011	49	Phil (94)	44 (31–60)	1.6	M (59)	30	Phil (97)	41 (32–50)	2.3	uHD (57)	L1_EAI2
ON267/BC021	41	Phil (100)	46 (36–57)	1.1	M (56)	20	Phil (100)	38 (30–51)	0.5	M/uHD (85)	L1_EAI2
ON155/BC187	26	China (54)	61 (41–76)	1.0	M (58)	15	China (79)	66 (50–88)	0.7	uHD (60)	L2_Beijing
ON150/BC038	18	China (61)	58 (37–80)	0.6	M (56)	10	China (100)	79 (73–82)	1.5	M (60)	L2_Beijing
ON181/BC046	20	India (65)	52 (33–71)	1.2	uHD (55)	9	India (78)	70 (56–75)	1.2	uHD (89)	L3_CAS
ON012/BC141	19	EAf (74)	32 (23–46)	0.6	M (68)	5	EAf (80)	39 (31–42)	1.5	uHD (60)	L3_CAS
ON058/–	13	SAs (69)	32 (22–44)	1.4	M (77)	1	SAs (100)	NA§	NA§	R (100)	L2_Beijing
ON104/BC157	12	EAf/EAs (75)	36 (26–52)	3.0	M/uHD (83)	4	EAf/EAs (75)	44 (27–59)	0.3	M/uHD (100)	L2_Beijing
ON179/BC149	9	India (67)	54 (43–75)	1.2	M/uHD (78)	10	India (90)	61 (34–76)	0.7	uHD (70)	L3_CAS
Intraprovincial Clusters											
Canadian-born											
ON219	15	Canada (73)	47 (34–62)	6.5	M/uHD (87)	0	–	–	–	–	L4
ON22	14	Canada (77)	56 (49–64)	13.0	M (64)	0	–	–	–	–	L4
BC001	0	–	–	–	–	28	Canada (96)	44 (33–52)	1.6	R (86)	L4_H3
BC003	0	–	–	–	–	26	Canada (96)	45 (29–52)	2.2	R (85)	L4
BC008	0	–	–	–	–	21	Canada (86)	47 (36–56)	0.8	uHD (81)	L4_Ural1
Non-Canadian-born											
ON73	11	EAf (56)	49 (24–57)	2.7	M/uHD (73)	0	–	–	–	–	L3_CAS

Abbreviations: IQR—interquartile range; L1—Lineage 4, L2—Lineage 2; L3—Lineage 3; L4—Lineage 4; EAf—East Africa; EAs—East Asia; SAs—South Asia; Phil—Philippines; M—metro; uHD—urban high-density; uMD—urban moderate-density; R—rural/remote.

* Predominant (≥50%) birthplace country or region. Birthplace was unknown for 10 individuals; percentage represents those with complete data.

†Predominant (≥50%) community type.

‡All individuals were male.

§Not available, data suppressed due to small cell size.

## Discussion

This study describes the first comprehensive interprovincial comparison of MIRU-VNTR genotyping in Canada using >4,900 *Mtb* isolates collected in Ontario and BC over a seven-year period. This represents >50% of culture-positive TB cases diagnosed in Canada during this period, and provides new insights into the comparative epidemiology of TB in two of Canada’s largest provinces. Although both provinces have large, diverse populations, there were significant differences in the epidemiology and the *Mtb* population structure between the two provinces. Ontario had more unique genotypes, primarily in persons born outside Canada, and more cases occurring in large urban areas.

Despite the high strain diversity, the clustered proportion differed significantly between Ontario and BC–similar to findings in a Western Canada study using restriction fragment length polymorphism (RFLP) genotyping where clustering varied (9%–64%) across the provinces studied [22]. In our study, BC cases were more frequently clustered than those in Ontario, consistent with BC’s higher proportion of TB in Canadian-born persons, amongst whom local transmission is likely to drive TB rates. This was further supported by the higher proportion of respiratory disease seen in BC, which is more common in those with L4 strains [1,23]–the lineage which was commonly associated with the large predominantly Canadian-born clusters. Encouragingly, most clusters were small, with only seven large outbreaks consistent with local transmission–most of which have been previously described [6,21,24,25]. Thus, despite different models of TB management and care between the provinces–Ontario follows a decentralized model and BC a largely centralized system–common practices and national guidelines [4] result in consistently effective public health responses in most cases.

When we examined genotypes present in both provinces, we found that most genotype matches were due to a single individual in either province–the vast majority were born outside Canada which is consistent with the notion that these represent LTBI reactivation [26]. There appears to be little interprovincial transmission between Ontario and BC, and the seven cases detected are genotypic matches to two strains endemic to BC circulating within vulnerable populations with known risk factors, including under-housing [1,21,24]. It is possible that these Ontario residents had travelled to or had prior residence in BC, with social/behavioural risk factors linked to a higher risk of exposure and infection–something that has been observed in other cross-jurisdictional studies [27–29]. Interestingly, Ontario’s large clusters (ON219, ON22) circulating amongst under-housed individuals in a Metro area of Ontario [6,25] are not found in BC, suggesting differences in the epidemiology or movements of the under-housed populations between the provinces. However, because TB case management occurs at the provincial/territorial level, sharing of patient-level data across jurisdictions is challenging, and a limited data-sharing agreement with stringent privacy requirements prevented comparison of risk factor and epidemiological data to explore this further. We also assumed that identical genotypes amongst Canadian-born individuals with L4 *Mtb* strains represented local transmission. This observation is supported by recent work in the English Midlands, [20] but whether this is the case here remains to be seen; it is possible these interprovincial clusters represent a common strain circulating in Canada amongst vulnerable populations. We similarly assumed that persons born outside Canada having *Mtb* isolates with lineages commonly associated with their birthplace and genotypically clustering across the two provinces were likely to represent LTBI reactivations with a genotype common to their country of origin.

The strong phylogeographic structure of *Mtb* [30] was apparent in our study. Lineages were consistent with birthplace and mirrored Canada’s demographics, where the top three countries of origin of immigrants to Canada are Philippines, India, and China [31]. These were reflected in the considerable number of L1_EA2, L3_CAS, and L2_Beijing strains and large MIRU-VNTR clusters within these lineages. It has previously been determined that the discriminatory power of MIRU-VNTR is reduced in non-L4 strains [32], and several studies have found that MIRU-VNTR overestimates transmission [2,33,34]. Our findings support this, particularly amongst persons from the Philippines–nearly all *Mtb* isolates from Filipino-born individuals belonged to sub-lineage L1_EA2, which is in line with recent studies [35,36]. The same two MIRU-VNTR patterns dominated within L1_EA2 in both provinces, and understanding that these represented common genotypes in persons from the Philippines could have prevented a significant amount of public health resources used within each province to investigate these clusters–investigations which to date have not yielded epidemiological connections supporting local transmission.

Currently, there is no coordinated national molecular surveillance program for tuberculosis in Canada and genotyping data are not routinely shared across all provinces, precluding a nationwide molecular surveillance program of the type implemented in the United Kingdom, the Netherlands, and other comparable low-incidence settings [37,38]. While our analyses suggest minimal TB transmission between BC and Ontario, these are two geographically distant provinces–a similar study using geographically closer jurisdictions may tell a different story. A national molecular surveillance program is a complex undertaking, requiring coordinated and collaborative efforts by all provinces/territories for implementation, maintenance, support, and evaluation. Perhaps the largest challenge is acquiring funding to support a national program, particularly the necessary personnel required to carry out such an effort, as provincial public health budgets are already limited. Additional issues complicate the ability to access and analyze health data across provincial/territorial borders–data ownership, legal, ethical, and privacy concerns limit what jurisdictions may be willing or able to share, yet clinical and epidemiological data are required for meaningful interpretation of genotypic data [39]. Interpretation of these data requires molecular epidemiologists with a regional- and national-level understanding of TB epidemiology, and a suitable information technology platform to link genotyping and administrative data. Integration of data sources, even within provinces, requires significant resources for creating and curating databases and routinely linking data. In Ontario, the OUT-TB Web online platform is used to communicate case-level genotyping data across the province and could provide a template for a national system [5].

## Conclusions

While there was minimal evidence of cross-jurisdictional transmission in the present study, the comparison of TB molecular epidemiology between Ontario and BC furthered our understanding of local transmission and LTBI reactivation by providing context to the genotypes observed in each province. This information strengthens the collective understanding of genotypic clustering and how it can be used to support public health efforts in TB prevention–essential for program management and resource allocation, as local molecular epidemiology often informs contact investigation and other TB program activities. Our study contributes to the understanding of LTBI reactivation of infections acquired abroad, providing further evidence that genotyping does not always provide sufficient discriminatory power to exclude local transmission–information necessary for determining appropriate TB prevention strategies. Next steps could include expanding the analyses to other Canadian jurisdictions, and incorporating whole genome sequencing data–used prospectively and combined with epidemiological data, this technology will most certainly provide the clearest picture of TB epidemiology and more accurately quantify transmission versus LTBI reactivation, for which different preventative measures are needed.

## Supporting information

S1 Table Genotype (24-locus MIRU-VNTR) results, including intraprovincial genotype clustering* by size and frequency in Ontario and British Columbia, 2008–2014. (PDF)

S2 Table 24-locus MIRU-VNTR patterns for study isolates. (PDF)

S3 Table Multivariable analysis of factors associated with single and multi (≥2 isolates) contributors to an interprovincial 24-MIRU-VNTR cluster, Ontario and British Columbia 2008–2014. (PDF)

S1 Fig Venn diagram representing the number of unique and shared 24-locus MIRU-VNTR genotypes between Ontario and British Columbia, 2008–2014. (PDF)

S2 Fig Proportion of single contributors to an interprovincial cluster by province and birthplace. (PDF)

S3 Fig Distribution of *Mycobacterium tuberculosis* sub-lineages in Ontario and British Columbia (2008–2014) by patient continent or region of birth. Pies are scaled to the total number of isolates represented by each sub-lineage.
(PDF)

S4 Fig Epidemiological curve of two MIRU-VNTR genotype clusters known to represent local transmission in British Columbia. (PDF)
