# Supplementary material for: Universal genotyping reveals province-level differences in the molecular epidemiology of tuberculosis
Source: PLoS One. 2019 Apr 3;14(4):e0214870. doi: 10.1371/journal.pone.0214870 (PMC6447219; doi:10.1371/journal.pone.0214870)
Supplement: S1 Table — (PDF) [file pone.0214870.s001.pdf]

S1 Table. Genotype (24-locus MIRU-VNTR) results, including intraprovincial genotype clustering* by size and frequency in Ontario and British Columbia, 2008–2014.

Characteristic	Ontario<br>*n* (%)†	British Columbia<br>*n* (%)†
Isolates		
Unique genotype	2261 (68.2)	953 (59.5)
Clustered genotype	1053 (31.8)	649 (40.5)
Clusters		
Cluster Size		
2 persons	164 (56.7)	73 (54.9)
3 persons	56 (19.4)	23 (17.3)
4 persons	20 (6.9)	5 (3.8)
5–9 persons	38 (13.1)	22 (16.5)
10–29 persons	9 (3.1)	7 (5.3)
30–49 persons	2 (0.7)	2 (1.5)
≥50 persons	0 (0.0)	1 (0.8)

**Clusters: ≥ 2 cases that share an identical genotype.*
†*Percentages have been rounded and my not total 100%.*

S3 Table. Multivariable analysis of factors associated with single and multi (≥2 isolates) contributors to an interprovincial 24-MIRU-VNTR cluster, Ontario and British Columbia 2008–2014.

Characteristic	Multi vs. Single **OR (95%CI)**	Multi vs. Single **aOR* (95%CI)**
Age, years		
0-14	1.4 (0.3–6.3)	1.0 (0.2–4.8)
15-34	Reference	Reference
35-54	1.5 (1.0–2.3)	1.4 (0.9–2.2)
55–74	1.2 (0.8–1.8)	1.2 (0.8–1.9)
75+	1.0 (0.6–1.7)	1.3 (0.7–2.1)
Sex		
Female	Reference	Reference
Male	1.3 (0.9–1.7)	1.2 (0.8–1.6)
Province of residence		
Ontario	Reference	Reference
British Columbia	0.6 (0.5–0.9)	0.6 (0.4–0.8)
Community type		
Metro	1.9 (1.0–3.8)	2.7 (1.2–5.8)
Urban, high-density	1.2 (0.6–2.4)	1.9 (0.9–4.1)
Urban, moderate-density	1.7 (0.7–4.0)	2.2 (0.9–5.7)
Rural/Remote	Reference	Reference
Birthplace		
Canada	3.3 (1.9–6.0)	8.0 (3.8–16.6)
Outside Canada	Reference	Reference
Lineage		
L1	1.0 (0.6–1.6)	2.4 (1.4–4.2)
L2	0.9 (0.6–1.4)	2.0 (1.2–3.5)
L3	0.6 (0.4–1.0)	1.4 (0.8–2.6)
L4	Reference	Reference

Abbreviations: OR—odds ratio; CI—confidence interval; aOR—adjusted odds ratio.
**Adjusted for age, sex, province, community type, birthplace, lineage.*

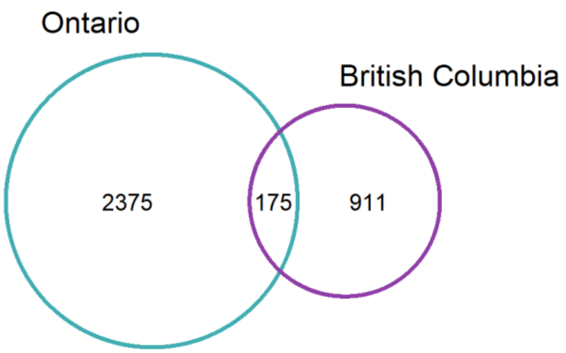

S1 Fig. Venn diagram representing the number of unique and shared 24-locus MIRU-VNTR genotypes between Ontario and British Columbia, 2008–2014.

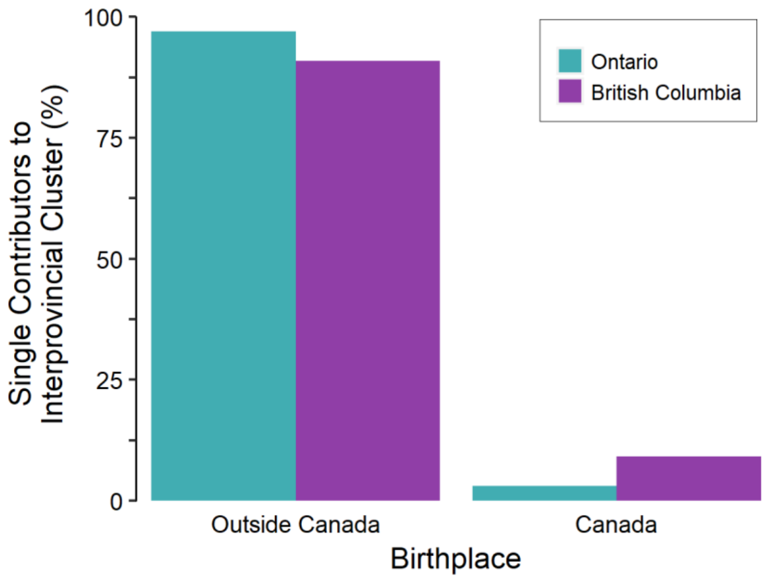

S2 Fig. Proportion of single contributors to an interprovincial cluster by province and birthplace, 2008–2014.

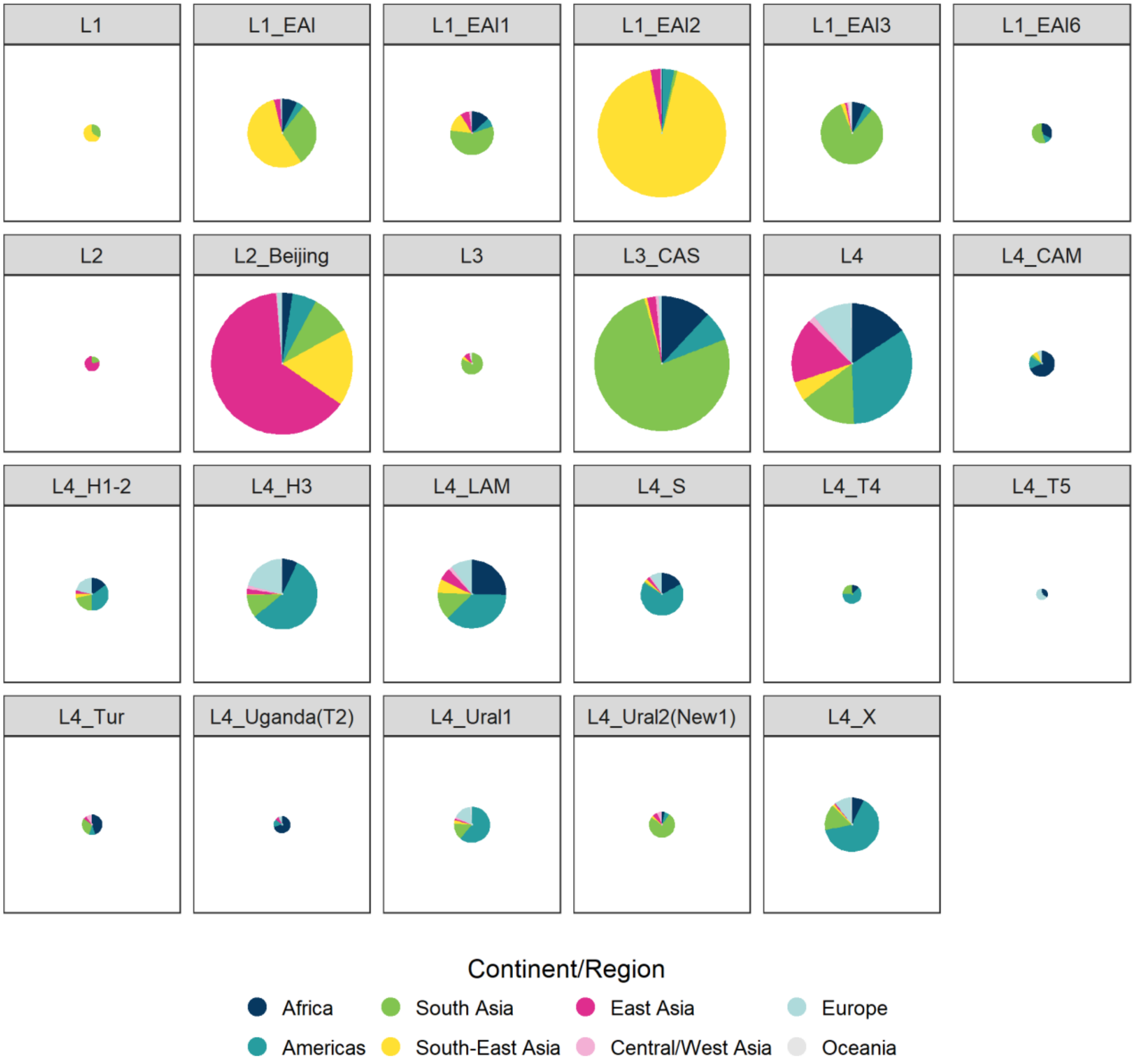

S3 Fig. Distribution of *Mycobacterium tuberculosis* sub-lineages in Ontario and British Columbia (2008–2014) by patient continent or region of birth. Pies are scaled to the total number of isolates represented by each sub-lineage.

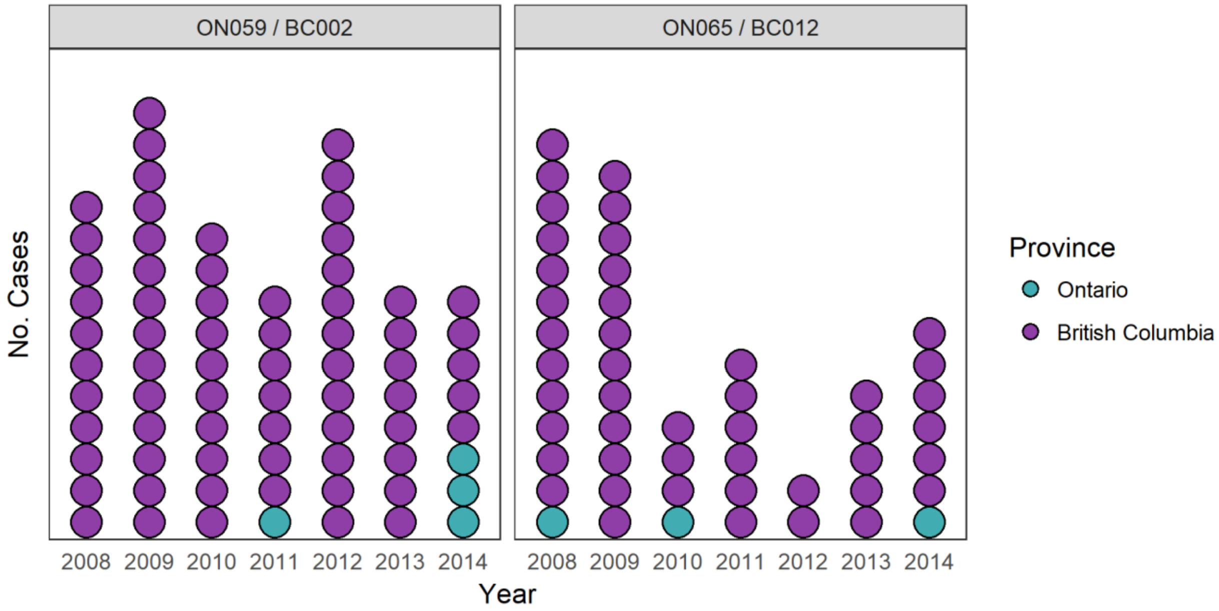

S4 Fig. Epidemiological curve of two MIRU-VNTR genotype clusters known to represent local transmission in British Columbia.
