# Supplementary material for: Universal genotyping reveals province-level differences in the molecular epidemiology of tuberculosis
Source: PLoS One. 2019 Apr 3;14(4):e0214870. doi: 10.1371/journal.pone.0214870 (PMC6447219; doi:10.1371/journal.pone.0214870)
Supplement: S2 Table — (PDF) [file pone.0214870.s002.pdf]

S2 Table. 24-locus MIRU-VNTR patterns for study isolates. Loci order: 580, 2996, 0802, 0960, 1644, 3192, 0424, 0577, 2165, 2401, 3690, 4156, 2163, 1955, 4052, 0154, 2531, 4348, 2059, 2687, 3007, 2347, 2461, 3171.

24-locus MIRU-VNTR Patterns
263233243232243252213412
273335424444748253213423
241313442122434262212415
353433444232415252213423
251333243272424262213413
273335544432658253213623
346333144232218252213423
5224341442216A7263223353
354333444212118252212422
253533233433335252213423
344333344232416252212423
5224341442218A7263223333
253433443433247252213423
373335444432557253213423
5224341422219A7263223373
212722344433236252213423
273335344432458253213424
5224341442219A7263223363
523335246251266253223343
5224341442219A7262223333
063335344442448253213423
083335444432558253213423
723436246161356263223313
212623344333134252113423
253433543433247252213423
622425247241256253223333
254532233433235152213423
8224341442217C6263123363
251313242242524262213423
272334344433459253213423
214744424224225253213423
273234444432657254213423
151333242242325252211423
273335444432598253213423
273545424224248253213423
253433233433536252213423
283745524234244253213423
212822344433237252213423
252432322122236152213423

24-locus MIRU-VNTR Patterns
253545524234248253213423
273335444432388253213423
522444144221987263223363
323225247241156253223343
1s224341442219A7262223363
143744424234248253213423
273235444432658253213423
346333344232218252213423
271325444431648253213423
243433234433437252213423
273335444432552253213423
5224331412219A7263223353
273335443232458253213423
273336444432658253213423
354233344232419252213423
353433344232414252113423
243223243232338252213413
273335444434945252213423
923435229221783261223343
252313243222425252113423
212822344444323825221343 3
251434443432443252213423
254432322122236162213423
5224341442214A7263223363
212723344233238252213423
623425246241252253223343
273335443232558254213423
253645323234247253213423
5224341442214B7263123363
623325247241256252223353
271645424234248263213423
522434144221997263233363
273545523234246253213423
254433433433338252213423
273335443432558253213423
274335344432658253213423
2224341442219A7263223323
4224341442219A7263223363
212822344443237252213433
5224341442211A8263223353
252133243232236252213433
263335244432558253213422
522434144221867272123353

24-locus MIRU-VNTR Patterns
272645823234248253213423
272335444432458253213422
524425246221252253223343
522434144221997263223343
273335444432657253213423
27333544443555A253213423
263645424234238253213423
255432342122226162213423
213722364433237252113423
253233242242257252213412
253533233433526252213423
522433144221987243223353
212513343433249252203423
253533133442527232113223
263335444432647253213423
212822344433247252213423
243335444432598253213423
211822344433238252213423
272335344433559253213422
274645424231257253213323
272335344433559253213423
255434433443339252213423
5224341242219A7263223363
252343222232125252213423
251533243222523262213423
251533233433637232113223
283665422434147233213423
273546324232228243213423
273335743232558253213423
273545324233248253213423
272435424234247252213423
5224341442218A4263223363
273645424234246253213423
3s223341542219A7262223363
523425246231251253223323
261423242122235262213423
273335344432548253213423
5224341442218A6262223363
251333243252425262213423
273644424444247252213423
273335443232557253213423
5224341442218A7262223363
324434246141363273223313

24-locus MIRU-VNTR Patterns
283335224433445253213423
273335444432569253213423
212513323433137252213423
5224341442217A7263223363
253645322234248253213423
263334444433647253213423
221423442122435211213415
251333243242524262213423
243333343232425262213433
273335444432558254213423
273435444432658253213423
252233242212218252212423
254423442222338262213423
232645424234248253213423
523635227221474271223343
273545524234246253213423
251413242122334262212415
25364542-234-45253213423
322434144221867263223363
251323232252423261213422
273545424234248253213423
5224541442219A7263223363
253645324234248253213423
274634424444247252213423
273335444432558253213423
522434144221857263223353
5124341442219A7163223363
214744424222235253213423
272334344433558252213423
11251324343323B252213423
283644424232246253213423
222335144221347213213423
252533233453537232113223
5224341442217C7263223373
25433224212223-262113423
273235444432658254213423
344333443232519252213423
5224341442218A7263223363
273335444444748254213423
652224344212-19252212422
5224331442219A7263223353
273545524234248252213421
263335244432558253213423

24-locus MIRU-VNTR Patterns
273335444432758253213423
251423441122438262113315
723436246161366263223313
5224341442218A7262221363
5224341442217A4263223363
7224441442215A5263223363
233432342212435262213421
271135444444647253213423
273546524234247252213423
623314247241256253223343
5224341442218A2263223343
173641224234248253213423
264333343212525252113423
273336433432648253213423
4224341442213A7263223363
272335444432658253213423
273335344434849243213423
245433233443547252213423
273635424234244253213423
273335-43442458253213423
273545524234245253213423
5222341442218A8263223353
212822344441238252213433
522534144221945263223363
251335444432558253211423
523434246241256253223353
354333344232519252213423
273235444432656254213423
523635227251775261223353
244433233423555252213423
273335444433658253213423
161413442122533262212415
212513143431238252213423
272345424234237253213424
294434442252325252212423
353433444232414252213423
254432322122237152213423
3s4343433345722625111 3423
523436246161366263223313
343336444432757253213423
531435244251A45273223343
263335544432458251213423
273335344432558253213423

24-locus MIRU-VNTR Patterns
322434144221887263223363
273335443412639253213423
243333233433449252213422
17454552-232-48253213423
5224341442218A8263223363
273335444432557253213423
522434144221A97263223363
273334454434747253213423
5224341442218A5263223363
273335444532658253213423
073335344422538252213423
273535524234218253213423
073335344432356253213423
27364442444424725221342-
283345344434742253213423
723426246251246253223343
233335245434244253213423
273335444432658253213423
1224341442218A7263223363
252645424234248253213423
5224331442215A7243223353
252432342122247132213423
254432342122255162213423
233645424234248253213423
273335444432458253213423
273335444432258253213423
343433444232415252213423
522434144221297263223363
5224341442218A7263223353
271335444432538253211423
623425246241256353223343
273545524234248253213423
273335432432658263213423
823436246161366263223313
273335444432678253213423
273335544432558255213423
522534144221887243223363
212744424224235253213423
246433342123239172113423
37433334423241A252213423
27354452-214-41253213423
254313243252222252213423
4224341442228A6262224363

24-locus MIRU-VNTR Patterns
5224241442218A7252223363
5224341442218C7263223363
623434246221286253223343
273344424444247252213423
253533233434237252213432
6224341442216A7263223363
273335343432458253213423
5234352442418-2261223343
623325246251276253223343
524434246141364273223313
524434246141366273223313
273235444434658253213422
522434144221697263223363
273335444332458253213423
273435424234247253213422
253545324234228252213423
251423542122438262213315
25352323-433-37252213423
5224341442216A7263223363
273646424234247253213423
522433144221957263223353
522434144221178263223363
242535224234248253213423
273644424442258253212423
373655523235245253313423
212513143433137252213423
522434144221987263223363
27334542-234-48253213423
523425245241255253223333
522435144221199263223363
261546524234246253213423
532434144221AD7263223363
523425247241256253223343
252443242232425252213422
234433442212336252213421
256233233443334252213423
26332544543466-253213413
273844424444247252213423
522434144221977263223363
27333544-432-57253213423
254433433413438251213423
273335444432658253113423
273335444434737253213423

24-locus MIRU-VNTR Patterns
523435246141364263223343
271335444432558253213423
283545522234248253213223
29353552-234-47253213423
292745-24234248253213423
271323342122438332213323
273345444432659253213423
261335444422558263211423
241432341122137273213423
272545424234238253213423
423435246161366263223323
424434246151367562223313
212822344433236252213423
1s224341452219A7262223363
2A1335544454746252213423
522434144221987263223323
353333444232415252213423
252533233423535252213423
273335444432688253213423
273335444432-58253213423
27333544443255-253213423
251333242242427262212423
452233444212515242212423
274235444432458254213423
252433144242233232213422
243335242423648253213423
273335443432658253213423
212513343433138252213423
272644823234228252213423
253533233433537252213423
252533233423435252213423
273645424234248253213423
214722354433237252213423
27333514443265A253213423
271335344413639252213423
27333544-442-38253213423
522434144221884262223363
25364542423624A253213423
264335444432498253213423
273644324444247252213423
273335444432638253213423
623425247241256353223343
723436246171366263223313

24-locus MIRU-VNTR Patterns
273232344432658253213423
273334443432554253213423
273335424432668253213423
522434144221887243223363
1234351262514B5261223344
5s53433443232419232213423
423435229261483271220343
271645424234258253213423
274335444432558253213423
5223341442216A6263223363
273136444444748253213423
273335444244648253213423
254243143222226252213313
273335444434548252214423
213513143433237252213423
422426235241654263221323
273644424230247253213423
241323442123424262212423
5224441442219A7262223363
212722354433235252213423
274-43424234248253213423
252344232232225252213423
212513343453138252213421
272344544434548253213323
273235444432458253213423
251333243232224262213423
422435247241563263221343
263545524233248253213423
524515246141366263223313
264634424444247252213423
5224341542219A7262223341
273334444430658253213423
273245444442656254213423
5224341442218A7263223343
444333244232537252213423
273635424234228253213424
273335342433549253213423
273645524234246253213423
251533233433337232113223
253233243231338252213313
5224341442217A72632233A3
424433247141365272223313
274644424444247252213423

24-locus MIRU-VNTR Patterns
5224341442219A5263223363
423345248231A66283233353
26333534443255-253213423
273335444432659264213423
623425247241255253223343
5224341442213-7263223343
253233242232247252213412
273335444432655253213423
273335344433648255213423
522426247221547262223363
5224341242219A7262223363
284645224234267263213422
522434144221AA7263223363
274325444432658253213423
273235444432556254213423
243323233222235252211423
273565524234248252213221
25443314343234-252213423
273535524232228253213413
273335444442649253213423
26354552-234-48253213423
523635229231764261133343
263345426244248253213423
263335444432658253213423
273545424224246253213423
523437329231684281223343
27354552623424-253213423
522434144221188263223373
363545524234247253213423
253213243232236252213413
212512343433236252213423
522434144221997263223363
623436245141256262223343
273546524234248253213423
256333243232325262213423
1s224341452219A5262223363
251335346433649253213424
453233544212515252212423
523425246141354263223343
522435236141366262223343
25364532423224-253213423
273545526234248253213423
273335345432549253213435

24-locus MIRU-VNTR Patterns
37433334423241-252213423
273655424234248253213423
273335443432558223213423
272644823234248252213423
253433243443448252213423
27333544-434747253213423
083334244432557253213423
273235444432558254213423
5225341442218A4263223363
232243242232325252212423
5214341442219A7263223363
27333544-432658253213423
322434144221865263223363
243644224232258253213423
273644424424247252213422
5224341342219A7263223363
272724224232245252213423
523635229261754271223353
45223334421261-252212423
271335444432555253213423
5224341442219A6262223363
5222341442213A4263223343
523433229221884221223353
5224331442219A7243223353
512234144221997263223353
424434246141-66262223313
273645423234243253213423
5214341442219A7263223343
253633231433337252113423
252333242232423252203423
2221341442218A7263223363
5225341442218A7263223363
522433144221895262223363
5225341442218A5263223363
273325444432488253213423
24354542-234-48262213423
273545524234248253211423
273335444432658253213723
26364542-234-47253213423
297433242232237252213423
253333243232325262213423
363545524225242253213423
252333242232215262213423

24-locus MIRU-VNTR Patterns
274335444432658253213423
252335444432658253213423
253533233433346252213423
27333544443474A253213413
283545524234248253213423
5214341442218A7263223353
252233343232344252213413
523425244261266253223343
34432334423231A352213422
272645424434245253213423
27354542-233-45253213423
272335254423648253213423
283335444432658253213423
29264582323424-253213423
343233543222316252213423
263545524244238253213424
273235444442656254213423
5224341442219A7262223343
522434144221787263223363
273645424434248253213423
252313242252425242213423
252313243222223252213423
525435246141366263223343
522444144221957262221363
5234331442219A7264223353
526315246141246263223313
522434144221887263223363
352332344222518352112423
273235444452659253213423
273331444432458253213423
522434144211977262223333
273545224211248253213423
5224341342218A7263233363
253233253232-26252212413
273335444432686253211423
522434144221857263223363
22333324321241A252212423
2B3335444532653253213423
271335444434548253213423
272335444432658253212423
524434246121386273223313
721436144221977263223363
522434144221A95363213353

24-locus MIRU-VNTR Patterns
253644324234248253213423
323525246221172253223343
243334354434747253213423
25442342221243-262213421
272335344433539253213423
2125133434331382-2213423
263335244432687253213423
322434174221867263223363
254533233433527252213423
623325247241246253223343
5224441442218A5263223363
263553424434243253213423
522434144221148263223363
273545524224249244213423
163335444432658253213423
274444424444247252213423
252433243443249252213423
354333344232518252213423
522435245141376273223343
254433533433233252213423
273335444432358233213423
153534233433336252213423
2s63334334414749242213423
273335243432558253213423
272334444432458253213423
5224341422219A7263223383
233545424234248253213423
173335444432637253213423
151323243252325262213422
5224341442218B7263223333
5224341432219A7263223363
271335444432558253211423
244213243242425262213423
253433143443449252213423
245223343232323262213423
253233243232257252213412
271645324234248252213423
233335444432558253213423
273325444432558253213423
5224341442218B7263223363
273633424235228253213423
253533333433336252213423
523435249241656243223353

24-locus MIRU-VNTR Patterns
2s63335442244347253213423
273235444442656253213423
272645823234228253213423
263335444432688253213423
27365322323413-253213423
24364532423-247253212423
523435229251694261223352
273545424432248253213423
273545523234244262213423
212413143433137251213423
283335444432655253213423
253335244433648253213423
263335444432658263213423
264525424431248253213423
273334444434648253213423
273335344432658253213423
273335444442656253213423
3s73435442233367253213423
523436246191366263223313
522434144221987263213323
5224331442218A7263223353
252343242242424252213423
27382444-211-4-253213433
271335444232559253213423
212822344433235252213423
252343242232423252203423
2A384442-433-4A153-13423
274335444432458253213423
244443433433338252213423
351333346212418252212423
273335343432658253213423
2s5433334423241A252213423
5224331442219A5263224353
5224341442217E5263223353
5224351442219A7262223363
7224341442218A6263223363
263633333432327232113223
522434144221996273223363
233545324234248253213423
5234342432418-7261223373
242313243252324251213423
273545524234234262213423
5224341442219C5262223363

24-locus MIRU-VNTR Patterns
5224341442218B4263223363
5224341442219A7263123363
242324154222336252213423
262645324224266253213423
2s63233243232318252211413
214432432212347262213421
212513343433136252213423
513435247241775262123353
233335344432558253213423
274335443442558253213423
261335444432658253213423
25364532-234-38253213423
5224331442219A7263223363
5224341442219-7263223363
5224331442219C7263223353
5224341242219A726-223363
623425247241255353223343
5223341442219B7262223363
5224341442211A8263223363
273235444453658254213423
5224341442219A5262223323
253532233433-34252213423
6224341442219A7263223363
263335444432698253213423
273235444442556254213423
3735-5424444248253213423
252343242232424252213423
273335453232567253213423
5224341442215A7263223363
45223244-212-14252212423
273335444422559253213423
5225341432218A9263223363
274665422434247213213423
273335344432548253213433
322534164221865263223363
273745224234247253113423
243545424234248253213623
274545523232-48253213423
263545424234248253213423
253223243232338252213413
721425247291246253223343
253523233433537252113423
27332544-432657253213423

24-locus MIRU-VNTR Patterns
252343342232226252213423
252633233231335252213423
27366442243424-213213423
522434144221887223223363
243845424244243253213423
273644424232216253213423
525434246161366263223313
522443144221997263223353
27323544443265B254213423
5234352292513B4261234343
522434144221986263223363
422434144221884263223363
28333544443486C251213423
5224341442216A7263223333
263335444432588252213423
263633524434248253213423
27354512-234-48253213423
27354652423423B253214423
27354252-232-51253213423
233645323233248253213424
263545424232248253213423
243345424234248253213423
272645823234247252213423
252333243232423252213422
253233241232439252213413
273535424234248253213423
252312242252322252213423
273645424234257253213423
212513343453137252213423
253335444432258253213423
522434144221995263223363
5224341442218A7262223353
453433443232419232213423
273525524244231253214423
254333243232325262113422
273335444442558253213423
522434144221996262223343
272645823234248252213423
273548524244246253213413
5224341442218A7263123363
522434144221857263223343
5224341442218A6243223363
353433443232419232213423

24-locus MIRU-VNTR Patterns
5214341442219A3262223363
522-341442218A7263223343
62342524B221E85271223323
5224331442219B5263223353
253635424244278253213423
273235444432656254213223
344433433433328252213423
273235444432558253213423
523535228251-74261223343
253645424234248253213423
252213243242323252213323
522425246141353262223343
522533144221887263223363
623525247241255253223343
273-43424232248252213423
273335444434648253213423
273355443432488253213423
25274542-234236253213423
25333544443264-253213423
273335044434438253211422
253235444432558254213423
263335444432558243213423
522434142221977263223363
423445248231966282223343
523425246221296253123343
263335444432558253213423
254313243252225252213423
27333234443363-253213423
622425247241254253223343
274545524235248253213423
4244352292513B4261234343
273234444432558253213423
523635228251674261223353
252435223234248253213423
273745424224248253113423
45222344-212214252212423
283335444432657253213423
273336444432657253213423
722434144221965273223363
5224341442219A7263224363
273135244434538253213423
251343243262424262213323
273335444443659253213423

24-locus MIRU-VNTR Patterns
522434144221DA7262223363
453433443232319232212423
5234352262414-2261223343
253433441212438162213421
34433234323241A252213422
623425247241256253223353
273333244423648253213423
623425247241253353223343
524315246161363263223313
272335444434547253213423
3224341442218A7263223363
273334444432568253213423
251335444332559253213423
45223344-212-14252212423
263312243232325252213423
252213242282425252213423
273325244434758253213423
284645423234248253213423
283336444233349213213223
25132314212-234262213523
254645324230248253213423
5224341442219A8263223363
374333344232419252213423
923425247241256363223343
251413442122438262212415
253433233443637252213423
254433233432527222113223
273235544432358253213423
453433343232419232213423
253533233413535252213423
273824445211847253213433
253645324232248253213423
623425247241256343223313
263335444432669253213423
272235444432558253213423
273335344432458253213423
253223343232338262213413
263335543434743253213423
272334344433559253213423
512435227251764261223323
354233334232415252213423
254433322122235152213423
254233243232336242213413

24-locus MIRU-VNTR Patterns
214744424224235253213423
273335244432658253213423
252213242282325252213423
245433423423437252213423
212413353443139252213423
27333834-433-49253213423
273545224234246253213423
824334146161364263223313
233545424234148253203423
273333544432458255213423
273335444434737252213423
112513443433238252213421
3534334442-2415252213423
27123544353255A253211423
5224341442219B5263223343
273335444412654253213423
253233242232216252213513
245433433433231252212423
241313442132533262212415
442233344212418252212424
623325246241226253223343
251323142122234262213523
242333243222325252213423
233335243433248253213423
233335344474847253214423
233335244413248253213423
233335344464748253213423
273644423445244252213423
425434246141362262223313
522434144221685243223363
623525247261256253223363
253233243232439252114413
363745124234248253213423
253433233433437252113423
522234143221995263223353
273335444434735253213423
353433444232415252213323
353433244232415252213423
173545524232248253213423
5224341442218A7263223373
27354552-234-4-253213423
27432444443474-253213423
273335442432735253213423

24-locus MIRU-VNTR Patterns
254223243232338252213413
253433233431344252214423
623425246241256315223343
452233444212217252212423
254232243242337212213413
723245247241866263223313
5224341442214A7262223463
152363343262223152212432
35433334423241-252213423
343333444232415252213423
241443442112137262212323
254432322122236152213423
278332342122234272213423
241333243242424262213423
273545424234247253213423
251423241122538262213423
273644423444247252213423
26354452-234228253213423
273335444542658253213423
356333344232517252213422
273334444432658273213423
5224361442219A7253223363
252313243212125253213423
271335444432558252211423
523425247241255252223342
23364542-234248263213423
524434245141374273223313
282555424432243253213423
27376432-434-46253213434
344333344232416252213423
273335344442558253213413
473335444432658253213423
253523233431537252213423
252434333433437252213422
27344552-234-48253213423
452243244212418252212423
243333344232336253213423
273235444432557254213423
5234352292513-4261234353
523425245241256253223333
273545524234148253213423
251333242252525262213423
273335443434648253213423

24-locus MIRU-VNTR Patterns
263233243232237252213213
243132244252337252213423
273335543432558253213423
254645424234242253213423
423432249271666263221343
354233344232417252213423
254313243242325252213423
253532233433535252213423
273346444432655253213423
2534334422124472A2213421
212513143433237252213423
924236246141363263223313
252343242232325252213423
355433444232217252213423
247632342122236162213423
232333141232325252213423
27322324223223-262211213
263834233473266252213423
273335444432659253213423
273555524234247253213423
723425246241266353223343
273135344434748253213423
5224341442218A6263223363
271635424234231253213423
252433243443349252213423
5234341442218A7263223363
252533233452426232113223
263236444432658253213423
5224341242219A7263223364
254323243232425252214423
45223304421251-252212423
522434144221967262223363
273245444432536254213422
214744424221235253213423
053335444432558253213423
25343344342224-252213423
273335444432747253213423
273355524224237253213423
251533233433537232113223
212513143433227252113423
253335444412658253213423
723425246241156253223343
273234444432555253213423

24-locus MIRU-VNTR Patterns
243335444432658253213423
274635524234238253213423
37333544-432-58253213423
252433543413248252213423
243232143232336252213413
4224341442214B7263223363
223233242232257252213412
273335444432438253213415
5224341442218A6282223363
2A1335444454746252213423
273335444444735253213423
35323344-211-15252212413
273335444444668253213423
233233243232227252213413
271335344433639252213423
263233243262134252213413
272645424235248253213423
223545324234238253213423
522431144221685243223363
423435246141266263223343
253333342232626252213423
5225341442215A7263223373
273335344444348253213423
273335344422428253213423
252342242232125252213423
284335444432658253213423
273335434432658253213423
24132344212233-262213412
273234444432658254213423
23334526434464-153214422
273544524234243253113323
251333243231524262213423
253533233423437242213223
5224341442219A7262223363
273545524264248253213423
424434245141363263223313
253533233413537252213423
254533233433335252213423
273545-22234248475213423
254233343433356252211423
253223243231342252113313
254333433433448252213422
5224331442219A7263223373

24-locus MIRU-VNTR Patterns
233332244432248253213423
273345344434742253213423
524434246141366263223313
271335444422558263211423
522435248241366263123353
25433444-434847253213423
244532243232-23262212523
452233344212519252212423
27333526444364B253213223
273235244432258254213423
322425247231256253223343
251333142242425262213423
274235444432656254213423
5224341442219A-262223353
522435246141363262223343
522434144221957263223333
243333244232237252213423
271335444432948253211423
25433324-252423252212423
272335444432558254213423
253655324232145253213423
273355424234248253213423
253533233431636252213423
273335544442558255213423
252343242232525252212423
272345444432468253213423
273635524234248253213423
723425247241267253223333
212513343433137252213421
254533233432626232113223
522434144221885263223363
241233242232-3-252213412
253533233432437232213223
272338344433539253213423
27264542-234-25252213423
-24435246141266263223313
273235444432636254213423
241323243250525262213423
251645323240148253213423
27333444-434548253213423
27333554443235-253213423
253333233433636152213423
44223244-212-15252212423

24-locus MIRU-VNTR Patterns
522434144221997262223363
27214532323424-253213423
212613342433135252213423
283644424221258253213423
252333242232425252213423
273545524224248253213423
27333544443474A253213423
252334243232624252213423
254223243231334252213413
623425247241256363223343
283545523234144253213423
283335443432458253213423
253335444432658253213423
12243414422174-263223363
7224341442218A7263223363
244422533433332252213423
294645422234248253213423
253535424233248252213423
223546524232238253213423
5224341442218A5282223363
263533233433636252212423
5224241442219A7262223363
263235444442636254213423
37433234423241A252213423
244646124234227253213423
27333544324334-263213423
5214341442219C7263223363
423435237221764263221342
274645424234267273213423
271335544434546253213423
522434144221DB7263223363
4224341442219A7262223353
5224141442219A7263223333
254433243232225222213423
27363542423424-253213423
256432242122224162213422
5224341442218A7273223363
283435444434648253213423
273745424234245352213422
254233433434428252213422
271335344433648253213423
273335444432658 25-213423
522434144221797263223363

24-locus MIRU-VNTR Patterns
251413442122437262212415
3s7133534443364825321342З
274644524442247252213423
523633229251775261223353
241334264212235252113413
281645324232228253213423
27364512-234-48253212423
522434144221894263223363
321434144221875263223363
6234352461613962B3223343
354333243232325262213423
524436145141365263223313
273635424234246263213323
524433246141366263223313
251645424234254253213423
253533233433634252213423
263337444432657253213423
212513143433137252113423
251423542122338262213315
293645424444248253211423
374333344232318252213423
273645424234244253213423
252432342122235162213423
263335444432559253213423
923435446141466263223342
273645424244247253213423
273335444234747253213423
522434144221997362223373
251333243242327262213423
252313243252125252213423
5224341442219A7263223353
B22334144221178263223363
233335244433258253213423
371335444422558263211423
293554424434243253213223
242433342113237162213423
273235445432658252213423
273331444452558253211413
652233343212516252212433
263335444432547253213423
223533233432636252213423
273744422432242233213423
273325344433248253213423

24-locus MIRU-VNTR Patterns
263335244432658253213423
244432342122136162213423
273745424234248253213423
273644324234248253213323
424434245141367212223313
283525524234248254213423
251334434432747254213423
123334344232238252113413
273645424434249253211423
153434233423746252213423
254313243262325252213423
273345424234248253213423
251333242252523272213423
274445424234249254213423
5234241442218C6263223363
243645524234241253213424
26354542423424-253213423
522334144221697262223363
243413242212437262214421
273544324234249253213423
27354532443224525-213424
353433324232216252213423
243344254232238252113413
623315144241256253223343
274333442432658263213423
344333434212414252112423
273545524234247253213423
273444224231212253213423
253522362122231162213423
523435249221757261233343
233335244432656253213423
27364542423424-252213423
523735229251773261223333
273445524234248223213423
253333242292434252113423
353533233433436252211423
5224341442219A4263223393
243233223232336252213413
254323243232524252113423
283553424434243253213423
423436246141347263223342
253533233433745252213423
252335444432657253213423

24-locus MIRU-VNTR Patterns
273335443442558253213423
5224331442218A7263221363
212513143433238252213423
253745324234228253213423
354333354232419252213423
27354542423424-253213523
273335444432657243213423
5224341442214A7263-23363
5234362461613C4263223343
273335464432548253213423
5224351442219A82432233A3
253533233443646242213423
253645324234247253213423
522435246141365262223343
253533233432627232113223
522434144221897263223363
251333243242325262213423
253445424436257253213423
332333244212518252212424
273545524234238253214423
273764322434246253213434
273545424234238253213423
5224341442219B7263223363
453233644212213252212423
243545524231248253213423
263654423244248253213423
273645524234248252213423
244335444432658253213423
255233242232228252213423
243545524434248253212423
274945424234245253213423
522435246141363262221343
251243243232423252213323
27133544442254A263211423
273323243232325252213423
524434242141266263223313
241313442122328262212413
244432322122236152213423
723425246241256253223343
523435245291A96263123343
452233444212719252212423
274645424234248253113423
251413442122431262212415

24-locus MIRU-VNTR Patterns
253523223433536252213423
253434443443227252213423
2533232432A2435252113423
254314243242423255213323
273645324234248253213423
273545524244248253213423
273335544432554255213423
524425249221575261223343
212513343433237242213423
252533233433527252213423
253333234232335252213423
274336444434447253113423
252353353262222152212432
273334444432448253213423
223335247433243253213423
244313233252424252213423
522434144221874263223363
253545324234247253213423
261432343423428252213413
273235344432548253213423
273335443442538253213423
253545524234245253213423
255433433631448252213423
292645424232238253213423
273434544432559253213423
273335444434537253113413
261423142122437262212423
5224341442219B6263223353
353533233433536252213423
214744524224235243112422
250432342122237162213423
265313243262225252213423
27364542-434-48353213423
243233244232236242113413
273644424444244252213423
252313243222424253213423
233645-24234244253213423
5224341442217B4263223363
253233243232259252213412
267433342123236162213423
2443132432B2326252213423
273545425431247253213423
273645424234238273213423

24-locus MIRU-VNTR Patterns
3s523432422322225252213423
5234352292516A4261223343
3s564323421222226262213423
283546524234248253213423
214744424224435253213423
254333144232332212213423
272335444432458253213423
271545524231248253213423
5224341442219A7263223373
312723444523238252213423
2433232432A2435252113423
523435249221775261223343
251413442122338262212414
523436226141366262223343
253445324234247253213423
253533232433337232213223
253325444432749253213423
522434144221997263223353
273545324234248253213423
7254252472412-6253223343
253533233433735252213423
253645324244247253213423
254432322122238162213423
273336244434247253113423
273135444434248254213423
522434144221797363223363
261745424232247253213423
263544524234244253213423
256333243232423252113423
354745324234227253213423
524434246141244273223312
623425247261256353223343
255312342252325252213423
25623324323213B232213413
5234341442216A7263223353
243645324232228253213423
273545524233248253213423
253645424214247253213423
253132243231-38252213413
353433444232415252113423
27133544443255825321-423
5224251442219A7263223353
233545424234249253213423

24-locus MIRU-VNTR Patterns
221645423234247253213422
252644421232247253213423
241513442122537262212215
073335344432558253213423
3s7333544443245825321342 3
5234352292316A4261223343
523435227251894261222343
321434144221877263223363
253533233433435252213423
523437226251784281223323
483445524234148252213421
263445524234248253213423
273645424234144253213423
263645524234248253213423
214513142433228252213423
342254252463632242213323
27363542-234-46253213422
214734324224235253213423
351423322122237262213417
273645422224248252213423
524434245141266485223313
282645823234246253213423
423435239241765263223333
6213341442219A7262223363
253645324234244253213423
5224341442219A7263223362
253845324234247253213423
423445246281667263221343
254333263252337252212423
253543233433326252213423
253335444432659253213423
322334134221997262223363
5224341442219A6263223363
28364532-234-4A253213423
524435246141365264223313
233645424234248253113423
273545524234248253113423
34274425-443632242213323
263545524234248253213423
273545424234248253213623
723436246191366263223313
5234352461413B62A3223343
271335444442458253211423

24-locus MIRU-VNTR Patterns
525434236141376263223313
251233243232436252213323
524335249141363263223313
2313332442H2635252113423
242313243252324252213423
26364542-431-48283211423
5224341542219A7262223163
273645424231243253213422
523425245241296253223343
214744324221235253213423
271335244432148253213423
241313243222227262213423
724434246141366263223313
5224331442219A7263123363
623425237251256253223373
263645324234247253213423
322334144221867263223363
253345323232-47253213423
28333544-432-58253213423
344333243242425252113423
253535324244248253213423
2-1432343423428252213413
5134341442216A7262223363
253335444432658253213623
422434144221984264223363
5234352461412C62A3223343
233646424234248252213423
152433223343349152213423
273645424224248252213423
253646324234241253213423
273545524234242252213421
244334254232235252113413
361333424252519252213423
253335444433646253213423
4224341A42214A6262223363
5234362461413D62A3223323
275332342120236272213423
273335444432558253113423
296433233423444252113423
522434144221967263223363
523435229241384261223343
253323223232227252213422
1s5223224323233825221341 3

24-locus MIRU-VNTR Patterns
322434142221867263223363
253223243232324262213423
424434246141377372223313
27333544443255A253213422
273335444442658253213423
522335236141363262223363
253635324234247253213423
273335464432658253213423
6224341442219B7262223363
273235444432659254213423
26333544-432-47253213423
322434144221867163223363
244433243453348252214423
231223143232-37252213413
253645324234228253213423
212513143434236252213423
273345324234149253213423
212513143433234252213423
213722354433236252213423
522434144221696262223363
253645324234246253213423
273335344432558253213413
273235344442258254213423
5234352392912C4261123323
422434174221877263223363
272345444432448253213423
422436249241B66263221343
52343622-251694261223353
35433334424241A252213422
213333232232524272213423
924435246141366263123313
253533233433337252212423
261645324234248253213423
523445226251684281223343
451233244212317252212423
232433341120237162213423
242353253232425252113423
253645324244248254213423
623425246241156253223333
272345344433448252213423
252343342232425252211423
244333243212328232213422
512434144221A97263223363

24-locus MIRU-VNTR Patterns
253645324224247253213423
253233242232426252213413
523433229261934261223343
273332444434647253213423
254432422122236152213423
21351344335313A252213423
522434144221867263223363
263623233432327232113223
255333243262325262213423
283824435211638252213423
212823325433138252213423
243334234232234252113412
212513143434237252213423
273634424234228253212223
2533332442-2635252113423
263335354222326252213423
824435256121266263223313
5224241442218A7263223363
257432342122235162113423
253533233343528232213223
273445224211248253213423
213513142431137252213421
723425247241256353223343
522435246141376263223313
2543232432-2335252113423
35233334423221B262213422
214743424224235253213423
273335442432658263213423
243323233433637152213423
214744424224234253213423
28354552423424D253213423
35433334423231A252213422
273226444432656254213423
254433432443438252213423
244334264232237252113413
522433144221997263223353
5224341442217A7264223363
251533333433637252213432
2s73545424234248253213423
273444324234238253213423
253655324234248253213423
243334234232234252113413
253233242242338252212413

24-locus MIRU-VNTR Patterns
273335454432658253213423
522434144221887262223363
21474452-224-35253112422
5224341442217E7263223363
254432142232423252212523
273235543432458253213423
243334264232237252113413
6224341442219B7263223363
252335444432558254213423
251233243232339252213212
622435249241665273223343
5222341442219A6263223353
251333243252424262213413
273545524235248253213423
273235344432558253213423
5234341442219A7262224363
273135444432649253213423
414425249261667263221342
5225341442217G7263223363
525336246141261243223312
242545224234248253213423
5225341442218A7273223333
253334254232236252213423
263725424234247253213413
243644224234258253213423
1s22435144221987262223363
524424246141364273223313
7223341442217A6263223363
525425249221C75261223343
263834233453267252213422
251213442122439262212415
283645423231248253213413
255333143262325262213421
453313444212415252212413
253533333433335252213423
263533233433436252213423
21474442-224235253213423
253313243232325252213423
243533233433735252213423
233223242232338252213413
234433442212437262213421
524434246141266263223313
2533332432G2435252113423

24-locus MIRU-VNTR Patterns
5224341442217A7263223353
252313243252425252213424
212513142433137252213423
214844424224235253213423
283335444232538253213423
5224341442219B8263213363
623424245241256243223343
5224341442218B6263223333
273645424234248252213423
253645324264247254213423
524334243141375263223313
253625324234247253213423
252313343232325262213423
512434144225CA7263122363
273335444432658253213323
271645424234245253213423
823425247261256253223323
254313243242323252213323
5225341442217B7263223363
274335444432678253213423
242312243222223252213423
271323442122335262213413
272635623234248253213422
522426147221546262223363
251423341122436262213315
273544324231249253213423
223233253232337252213413
273335344432656253213423
27434542-234-48252213423
27354542423424A253213423
251413442122538252212415
112413443443237252213423
D23436246171376263223313
422434144221987263223363
271335344432558253211423
221413442122435262212615
3s4433324324232525221342З
261323142122234262213523
251413432122338262112415
273335444432688252213423
223427246241258253223343
5214341442219A7223223363
253645326224128253213423

24-locus MIRU-VNTR Patterns
273333443432559253213423
283333134423648253213423
273335324433638253113423
2512332442F2235252113423
273745424234248253213323
624425247241234253223343
523633229251575261223352
233645424234244253213423
413513143443239252213423
253334444432258253213423
253533233332627232113223
252133343232236252213333
922425247241256253223343
33274425-443-32242213323
272334344431459253213423
252433243443339252213423
251213442122537262212415
522434144221867262223363
5224331442215A7263223353
522425247221787262223353
353433543242515252113423
5224341442218C7223223363
5224341422218A7263223363
623635227251774261223353
252625723234248253213423
214644424224235253213423
252533233433335252213323
5234252282913C4261223343
223335228241964253221323
25234423-232225252213423
252533543433326252213423
5244342461413A4263223313
283644424444247242212423
5224341442219D7262223353
256532333453547152114423
212513432413138251113423
253535444432659252213423
273644224444247252213423
324434236141376263223313
A22425246241255253223343
253232243232338252213413
523435245141366161223342
254433433443448252213423

24-locus MIRU-VNTR Patterns
273645324244238253213423
34433344423251A252213423
5224341442219A7263223343
271335444412554253211423
5224331442218A7262223353
271335444432559253211423
27333544-432-58253213423
344233343232415252213422
253655324234259253213422
253533233433637232213223
252543233433235252213423
623425252241256353223343
251333242252425262213423
353233243232257252213412
5224441442217A7263223363
5224341442212A7263123363
442233444212518252213423
523634229251774261223343
233333344232536252213423
273435424224247253213422
5224331442219A7213223363
27464642-234538252213423
523425243281266253223343
351213442122417262212413
5224341442219B7262223363
5224341442217A2263223363
521434144221967263223363
5224341442212A6262223363
245433233433549352213423
283335444432557253213423
25142354112233826221331-
27354542-234-58253213423
251313442122439262212415
273336443232558253213423
522434144221877263223363
214744424224433253213423
273545524234248244213423
355332344232414252212423
285323263242434252113423
251423442122337262213415
263646424234247253213423
273445524234248253213423
253746124234247253213423

24-locus MIRU-VNTR Patterns
5224241442316A7263223363
27333444443255C253213423
273335124234248252213423
5224351442219A4262223363
24164432423224825 2-13423
5225341442215A7263223363
252343242232425252113423
723425242271256253213353
6224341442218A7263223363
354332344232413251213422
423425247241257253223342
5224241442219A7263223363
273545524234224243213423
3214372292517B4261223333
236433342123239172113423
213513143423238252213423
254333243232324262213423
525435246141366264223343
273235444432648253213423
273335544432658253213423
425434246141336263123313
922435346141266262223343
243533232432627232113223
5224341442219A6252222363
523436246161356263223313
263745424232247253213423
422436249241965262221331
233445443474946153213423
273335244443548253213223
523435229251674261223343
273545-22234248253213423
263345444232658253213423
5224341422315B7263223354
272335444432468253213423
253724444211848223213433
274334444434737253213423
522435246141363262323343
35353323-433537252213423
5224341442219A7362223363
6224341442218B7273223363
273335343442558253213423
213513143433238252213423
271335244431549253213423

24-locus MIRU-VNTR Patterns
5224341442217A72632233-3
251333243252524262213423
273345444432658253213423
251413242122337262212415
622425247241275253223343
27333406543484B253213423
273535525234248253213423
241323142122434262213415
5224331442219A7253223363
263345444432658253213423
273336444432558253213423
254432342122236162213423
623324247241156253223343
251423542122439262213315
9224341442215A7263223363
324434246141357263223313
244432233433648252213423
5224341442213B7263223363
251313442122238282212415
273235454432558254213423
213722354433238252213423
242335454242237252213423
254533232433637252113423
362233244212515252212423
252533233431635252213423
212513143433237252214423
234335244433247253213423
271545524234248253213423
512434144221997263223363
521433144221997243223353
4224341442214A7263223363
524435246161326262223313
522426147221547262223362
253335444432549253213423
273554424434243253213423
522434144221996263223353
322244144221867263223363
214734422224435253213423
242343242232425252213423
524434246141364263223313
262335443432578253213423
254433443433338252213423
522434144221767273223363

24-locus MIRU-VNTR Patterns
253533233332626232113223
354333344232415252213422
523434246221AD5261223341
5236352292814A4271223343
264335443432548253213423
273335244443648253213223
5234352292613A4261223343
273445424232249233213323
27333534443245625-213424
242645424232248253213423
262433243433347252213423
233333243212325262213423
523635229291462261223353
522434144221767263223363
6s224341442214A6263223363
522434144221887263223343
274845324234247253213423
234313244272338252213423
5224341442219A8263123363
265433533433438252213422
2512332442-2634252113423
273235444432458254213423
273235444432668254213423
25364612423-247253213423
251213442122538262212415
253325444432648253211423
273644424444247252213422
322434134221997262223363
523435345141363263223343
5224341442216B7263223363
252533233433335252213423
5224341442219A7263323363
274645424234248253213423
622425247241256253223343
523425129271-84261223333
233333354232532253213423
254433443413345222213423
243233242232225261213423
522434144221D87263223383
233645326234547253213423
223433233433137252113423
263335444442669253213423
252533243242525252113423

24-locus MIRU-VNTR Patterns
333433244232519252213423
271645424234248253213423
243233243232437252213413
273314444432458253213423
273335444432558253214423
524424246141365273223313
5224341442217-8263223363
252432443442449252213423
622125247241246253223343
256433342123239172113423
143233243222231251213413
253523233433537232213223
252232323222525252213423
524434236141346263223313
522334326141363263224313
273-35444432-5625-213423
271335444422549263211423
253655324234247252213623
251423541122438262213315
5224341442218A6263223343
273544424444247252213423
522434144221967263223393
243232233232737252213413
6224231442219A7262223363
252422342122237162213423
243333344232536242113423
252532322122236162213423
34284325-443332242213323
623425245241256353223343
354331344232415252213423
24433344423251A252213423
253234342222338252113413
243333243232323252113423
272435324234247253213423
4s23435239241765263223333
522434144221876243223363
261432343423428251213423
423635229271774261223353
263532342122226162213423
254432322122226152213423
254343243232425252212423
273335543432548253213423
255423433433238252213423

24-locus MIRU-VNTR Patterns
823436244141366272223343
273236444442688253213423
2s2243414422176726322336З
522434144221A37263223263
253655324234247253213423
523433144221987243223353
5224331442219A6243223353
2A3335444432689253113423
4234361461413-6283223343
523426246241251253223343
522434144221987262223363
271345344443449253213423
522424144221997262223343
272325444434546253213423
251533233433237232113223
250432342122235162213423
253533233433534232213223
5234352282717H4261223343
263335344433446253213423
5223341442218A7263223363
243213142232428252213223
523424249231676243223343
255532233443437232213423
3224241A4221867273223363
210723144523238252213423
5224341422219B7263223373
45223324-212-14252212413
273635224232244253213433
252333242232225252213423
253533233422527232113223
352333244212518252212424
253332243232423252213423
35433334423251B252213423
253222243252439252213313
253533233432425232113223
243313243252324252213423
251413242122237262212415
293545424233247253213423
252342242232325252213423
2543232432A2335252113423
512434144221CA4263223363
152353342272324152212432
241433243463646252213423

24-locus MIRU-VNTR Patterns
5224341342219A7243223363
23832436A221B3-322162544
524424237141276263223313
271745424234245253213423
322434174221967263223363
273645224232156253213423
253334232432658253213423
212513143433138252213423
2634463244342-8253213423
273455424224248253213423
274235344432658254213423
274645524224248253213423
524434246141354263223313
623325247241256213223343
452233474212518252212423
253233243232336252213313
2533232432A2435252113422
5224341242218A7263223363
523432239241835263223333
255432342122235252113423
423425247241256353223343
212513243433137152213423
273336344432558253213413
623436246161366263223313
452233244212412252212423
272824445211749253213433
523425249221685261223333
523434229251694261223333
254333243232525252213423
2543132432-2324252213423
2543232432C2435252113423
5224341442219A7263213363
263233243222258252213412
263646124234247253213423
244313242272225252213433
5224441442219A7262223353
622434144221996263223363
5224341442216A5263223363
253233142232338252213413
273335444434738253213423
27354552-234-45251213423
293743524234245253213423
524434246141366263223213

24-locus MIRU-VNTR Patterns
257322242122237262212423
243232433433544252213412
522333346141393263223313
270645424234248253213423
283645543231248253213423
25233343321241B252212423
242545523234248253213423
522434144221695263223363
-73535524234238253213423
27333544443454A252213423
253233243232247252213412
271335444432558253217423
623225247241256253223343
263434233431334251213422
252333154242334232213422
272334344433558253213423
2422232--232336252213413
5234352292617C4261223323
422434124221987262223343
273545423234248253213423
423445248231965283223343
253533233433425252213423
26334652423424A253213423
233655424234244253213423
252343242212325251213423
253533333433335242213423
25243324343344C252213423
251333244242435252113423
322534144221867263223363
35433334424241C252213423
273335443232546253213423
254645122214248253213423
35632234423241C252213422
5224341442219A7263223333
262433233433545252213423
623524247241252253224343
5224441442219A7282223363
271335344433629253213423
271335444432568253211423
2533332442B2635252113423
256313243232422252213423
522433144221996243223353
273644424232247253213423

24-locus MIRU-VNTR Patterns
273335444432658254213423
623425247241246353223343
27354552-234-48253213423
351232143222-35052213413
271444424231247253212423
253445224231248253213423
252533233433436252213423
3234352292713C4261223343
522432144211985263223363
2s43433433433328252213423
353433444232419252213423
523425245241251253223343
522435144221897262223363
522434144221484263223363
293645223234244253213423
34274425-473-32242213323
422223344212318252212433
251413442122338272212415
252344242232525252213523
233533433433224252213423
264233242232337252213413
234654424234249253213423
273525624234248253213433
253333244232335252213323
263348423234249253213423
35223344-212-14242212423
253533233432527232113223
722334144221777263223363
254433443441445262213323
252422342122235162214423
6224341442219A7273223363
27353552-234-47253213423
253645324234241253213423
263444424444247252213423
273335444442659253213423
452645424234266253213423
524434244141366261223313
523435249221665261233343
251233243232435252213323
5233352222416C4261223333
452223444212715252212423
14274425-443-32242213313
523435246141366263223343

24-locus MIRU-VNTR Patterns
273645424231238253213423
273345524234248242213422
274534424454247252213423
244434233433338252213323
423425246251252253223343
623325247241246353223343
522434144221967262223343
273445423234147253213423
27333544343274B263213422
252333342123235161213423
5225341442219A7263223363
522444246141364263223313
62-425147241256253223343
252232343232526252213423
273674424444247252213423
271545424234241253213623
273335344433448252213423
253233243232247282213412
353232243232337252213413
272645823224248253213423
273335543432658253213423
27354552423424-253213423
273636424234248253213423
246433233433448252113423
273544524234233253113422
241423242132533262213415
522434144221885263223373
28354552-232-48253213423
27333544-432338253213423
21251314343323A252213423
253533333433537252213432
522436246141365262223343
9224341442218B4263223363
352332344232516352213423
254433233433538232213422
5224331442216C7263223383
522435225141347273223343
5224341442219A7263223313
524635229281474271223323
352332344232415352213423
35433334423251-252213423
42243724A241766263221343
283545323234248253213423

24-locus MIRU-VNTR Patterns
251332241262424262213423
523435229251774171223343
723425247271256253223343
263233243232247252213412
1s22434144221567263223363
153233241232337252213513
253533233433636232113223
252645723234248253213423
251422241122238262213423
522634144221787263223353
4234352451412A62A4222343
452233444212519252212423
275335444432358253213423
214734424224235253213423
273335444432656253213423
524315246141346263223313
723436245161365263223313
253433442212447262213421
274545624234248253213423
242353343232424152212442
258433342123236162223423
253433143463449252213423
5234252292516A4261223343
273335444432628253213423
273644524444247252213423
25653233345354D152114423
522434144221487262123363
623325247271255253223343
241323142122234262213523
262335444433559253213423
24354542423425-253213423
253353342252423152212442
424436246141366273223314
252433223463344252213423
253633233433626252213423
212723343423238252213413
355333344232417252213422
243333343232524122213423
523425249221C95261223323
274135544434655273213423
283554424434243253213423
273445424234238253213423
254644424444247252213423

24-locus MIRU-VNTR Patterns
253845524234248253213423
2632322432322-7252213412
351323344232419252213423
345334343232412252213423
251323142132234262213523
9224252442419-6261223363
253533333433437232213223
273335444432658253211423
622325247241256253223333
212413343433138252213423
4224352482419A7263221343
263545424224248253213423
5224341442218E7263223363
251423142122338262212423
273235444432452254213423
45323344-212-1-252212423
2523422B2222325252213423
251413442132439262212615
274335444432458253215423
522434144221997263223333
273235441432658251213423
34264425-443-32242213323
253532233432333252213423
42343524A241556263221343
173545524234244253213423
254533133433535252213423
263233243232257252213412
273355524224238253213423
523435229251563261223343
24843244-122214262113423
354313243232425252213423
252232444212717252212423
234434633434437252213423
522534144221887263223363
261645424233249253213621
5224441442219A8263223363
5224341342218A7263223363
354333344232517252213423
253233243242247252213412
5244352462414C3261223343
34174325-343552242213323
253533233433635252213423
5124341442218A6263223363

24-locus MIRU-VNTR Patterns
243335444434348253213423
354334344232517252213423
254313253242425252213423
2s5323324323233825221341 3
524325247281255254213343
622426242241256253223343
244233142232337252213413
273545123234448253213423
253333233423337252213422
273643424234248253213423
273545524234244252213423
273435424274247253213423
253645524234248253213423
273634424444247252213423
2733354-3232568253213423
263445424232248253213423
253533233433736262211423
254313243222325352213423
5224341442213A7263223363
422434144221897263223363
263635422432146233213423
352332344232419352213424
252133223242234251213433
241333243232423262213423
283435524234242253213423
273644424433258253213423
254433443433442252213423
263325444432647253213423
243433243433537252213423
34274225-464-32242213323
522426147221447262223353
26443323343343-252213423
354332344232517352213423
623325347241254253223343
27354552-234-47253211423
251423332122437262213414
5224341442219C6263223373
253343424234228253213423
273544324234245253213423
263824445211944253213433
524434246141356273223313
253533233433444252113423
524436246141366263223313

24-locus MIRU-VNTR Patterns
522435245141377273223333
42343423D241223254223343
27354552-234-4B253213423
273335424442559253213423
723636246161366263223313
273544524234243253113423
5124341422219A7263223363
253223243232217252213413
234333243232320262213423
263336444434546254213423
354213344232416252213423
253533233433535232213423
273545524244246253213423
273235444332658253213423
283235444432676254213423
283545524234248252213423
254233243232344252213412
27465532-234-48252213423
523635229271744271223353
273335443432458253213423
253533233433735252213422
123425247251256253223353
236433143123236162213423
273555424434145253213423
253333333433637152213423
27333544443265725-214423
27354542423424-253213423
255433233431348252213423
5224341442211A6263223363
212923444433238252213423
353645424434248253213424
283555424442243253213423
34274425-443-32242213323
252422322122236162213423
251645224234238263213423
253533233433536252213423
273337244432558253213423
323635228251674261223353
273334444432368253213423
253333243242325262213423
273334344432658253213423
267632342122236152213423
251646124233148253213423

24-locus MIRU-VNTR Patterns
452233444212418252222423
254343433431435252213423
522435225151378273223343
263555422434244253213423
5224341542218A7263223363
253533133433543252213423
284335443532548253213423
256433233431448251213423
254314242232325252213423
522435144221887263223363
263235444432646243213423
253233243252237252213313
3s53534433455447252213413
273644424247248252213423
246433342153237162213423
26441343345343B252213423
251333243252324262213413
274335344432558253213424
523435246151366263223343
241333142252425262213423
523435229251173261221343
243433233433648252213423
244322343242625252213423
27354552-234-4C253213423
243523233432327232113213
263335444432598253213423
251313442122333262212415
245433333433338252213423
623525247261245253123353
423225247241256253223342
253233243252337252213413
623435246241266253223343
524335246141366263223343
251645324233237253213423
273435443442558253213423
212313343433127252213423
2535332-3433526252213423
2742354434426-8254213423
272334444432658253213423
143333242232325262213423
251323243260524262212423
274645424234247253213423
34274525-443732242213323

24-locus MIRU-VNTR Patterns
294645424232246253213423
623424257241245253123343
212512374433136252213423
5224341542217A5263223363
273644424444246252213423
263334443434548253213423
253534233433736152213423
251323223232226252213423
253A75424444248253213423
27353542423422D252213423
234433233453537252203423
213513143433239252213423
234433442212435262213421
254432322121236152213423
27364542-234-38273213423
243213243232234252213314
254333243232422252213423
253223343232327252213413
273334444432758253213423
212A22244433238252213423
271335344443535252213423
272335443432458253213423
312724434533135252213423
253233242232137252213413
273235444432658264213423
273335544432568255213423
342743252443-32242113323
27133534443364A253213423
2513332422-2425262213423
173545524234248253213423
253433333433427232213223
173235444432556254213423
2735455-4234248243113423
523635229251775261223353
244532243232223262212523
244333242242433252213423
273545424224245253213423
253324154222237252213423
263335443432347253213423
252343242242525252213423
243334234232233252113413
253635424234268253213423
353533233433534252213423

24-locus MIRU-VNTR Patterns
273335444432457253213423
522434144221CC7263223362
523432246141366262223343
241413442122537262212216
273335444432658253213223
241333243252423262213413
246432342122236162213425
254345243232526252113423
523425249221C85261223333
822434144221971273223363
273443524232243252213423
5224341442214B9263223363
254313253233225262213423
273645423244248252213423
522534144211897263223353
524434246141266263123313
513438222251674261223343
243545524234248253213423
212514143433137252213423
251313442122237282212415
524434256141364263223313
244313243242423252213323
254533133433636252213423
24364532-234-48253213413
252533233432426232113223
073335444432537253213423
27354542423424B253213523
263745323244248253213423
214744424224635253213423
2513332442D2534252113423
250432342122236162213423
273334444452688253213423
273645224232256253213423
273334444432658252213423
241233243232436252213323
5124341442318A7263223363
253333244262535252113423
254533233443735252213523
273333444432558253213423
273644224232156253213423
522334144221887243223363
273645324224248252213423
5224331442219B7263223353

24-locus MIRU-VNTR Patterns
271645324234235253213423
273234444434648253213423
273235444442658253213423
523435227271735261223343
522334144221AF7263223353
273235464432648253213423
233333443212433262213421
422434246141377272223313
273335444412688253213423
273334445434847253213423
524434246141376273223313
212343222323222252113423
5234331442219A7263223353
273345444432559253213423
273234344434556253213423
522434144221467263223353
273234444432658253213423
253533232444437252213432
251333244242635252113423
273645522234249253213423
273235445432638252213423
2732353B3432548253213423
283244524434249253213423
252335354232327252213422
273645223235648253212423
253533233233337232213223
251322143232515262213423
251213442122237282212415
241423541122438262213315
5124341442219A5263223363
254313243272324252213423
273445524232248253213423
271335344433448263213423
275655424434249253213423
273555524234248253213423
523434229251373261234343
2638342334-3267252213422
273535524234238253213423
522434124221997263223363
242335254242327252213423
353433444232315252213423
252645324234256253213423
253333233433333252213424

24-locus MIRU-VNTR Patterns
27375432243464A253214423
242745424232248253213423
442233242212213252212423
2533232442B2335252113423
251312442120436262212414
27364522-235-48253212423
273645324234247253213423
272645324234148253213423
322434144221567263223363
253333234232336252213423
421445248231966284223333
234523433433224252213223
8232362461413A62A3223343
253433442443147252213423
26465542-234-48252213423
273134444434648253213423
423434246141346262223343
252335424432658253213423
353233243232425252213413
214744544224235253113423
27333544-432-59253213423
214745424224235253213423
245334254232237252113413
253655324434229253214423
244443333433445251213423
283625524434249253213423
273335444432478253213423
3234352292516B4261223363
163644424222257263213423
271645424234247253212423
273545224231259253213422
324336245141363263223313
263335444432678253213423
254432342122245162213423
273345344435742253213423
424436246141166263223313
252354343252424152202442
273945524234228253213423
254333243212325252113423
27364552423424825З-13423
254433333233438252113423
263745423233246253213423
273645424234249253213423

24-locus MIRU-VNTR Patterns
272645224234246253213423
254333244292244252113423
214734424224635253213423
523635228251884261223343
263533233432126232113223
255432342122336162213423
231335344454748253213423
212343222232422252113423
263335444432668253213423
273335444432468253213423
25753233345354C152114423
273645224231243253213423
2513332442J2535252113423
272645823234248253113423
272645424234244253213423
251233243222534252113423
253633233441435252213423
272336344433559252213323
21483442422-235225533221
273645424234238253213423
253524233433537252113423
273335344432357253213423
5224332442218A7263223353
423435249261666263221343
112513143433238252213423
273545424224248252213423
256432341122235162213423
522444144221957263223363
251413442122234262212415
273645424254258253213423
273237444432657254213423
272644424233148253213423
254433222433558252213423
473645424234247253213323
2513134421222 3A262212415
252433342123237162213423
3513333442 3221B262213423
273633424234248253213423
233645424234247253213323
723425247241236253223343
24374442-234848253213423
251232143232334252213313
263335444432659253213423

24-locus MIRU-VNTR Patterns
423335229241823261223362
5224331442219A7264123333
253434543433347252213423
523425249221C95261213323
271423541122436262213315
261423442122338262213415
223424244241266253223343
523435229251884261223343
273332444434647253113423
253335444432458253213413
523836226251284261223343
243232233433644252213413
354333344232416252213422
224323243232525252213423
253533233433436252213423
333333134232237252213323
55433334424241A252213423
273325344434648233213423
273235444432658254213422
252333242232325252213423
522434144221685263223363
523635322251-84261223343
273335444432559253213423
261333253242424262213423
262645224234246253213423
254533233433437252213432
272535324234248253213423
253643324244248254213423
522425247221587262123353
273334444432658253213423
212513143433136252213423
263645424234248253213423
273635223234247253213423
284434242262325252212423
273335444434747253213423
241433234433437252113423
283235444432659254213423
2433354A4432658253114423
523436246161377263223313
273335544432458253213423
273335243432453253213423
723445247241975262223313
253335444432558253213423

24-locus MIRU-VNTR Patterns
253533133433537232213223
524434246171366263223313
273335524234243253213423
623425524241246353223343
233532532433438252203423
252343242232225252213423
263213343232247252213412
212413343433248252203423
273425524234248253213423
2735455-4234248253213423
523615247221666263224363
354333344232312252213422
424435246141386263223344
34254425-443-32242213323
273645423424247253213423
213712354433235252213423
253335444432458253213423
254635324234247253213423
512434524221428263223363
242313243272324252213423
251333142232424262213423
272445324234238252213423
5224341442218-2263223363
522424144221967262223363
271423242122235262213413
254232342122237162213423
25264532-245-48253213423
253433243453348252214423
5234241442217A7262223363
123635229251765261123352
343332443232319252213423
254333333433339252213433
524434246141376263223313
5s234352292315C4261123343
254645424244248253212423
521426247221547262223353
357432343122236162213421
823424257241236254223343
521435229291354241223343
2A1334544454746252213423
251333243241424262213423
251423442122536262213414
524434246141366233224313

24-locus MIRU-VNTR Patterns
342243343212716252212423
273545223231238253213423
5224341422218A7263223373
257433342133337162213423
253675524433248253214423
522435246141373262223343
244223342232348252213413
272444424234247253213423
27364542224424A253213423
253335444432656253213423
3543333442325HA252213423
34174425-443-32242213323
253233243232338252213413
233545424224249253213423
251323142122233262213523
263725424232258253213423
254433433434438252213422
273335442432667253213423
254345324234248253213423
255533333433537252214423
262644923234248253213423
273635224254245252213423
273645624224236253213423
423425248221C25261223353
522434144221877263223343
252344132232225252213423
251644326234236253213423
252313242252424252213523
473335344433549253213423
261323143122234262213523
281645424234238253213423
243432233443642252213423
252432142232423252212523
242313243242224252213423
522425247241256252223343
273335544432538253213423
254433233433527252213423
243645424234258253213423
2B3335424234248253113423
273335444432568253213423
212513143433239252213423
212723344433138252213421
233345344432458253213423

24-locus MIRU-VNTR Patterns
524434246141366264223313
255233342121136162213423
273545524234248253-13423
524435246151366263223313
273234445432638252213423
112513143433137252213423
522433144221577263223353
7224341442219A7262223363
243432341122235152113422
523335248271263264221343
263645434233248253213422
245334154232234252113413
273335444432658253213432
212513343433248152203323
234313243282325252213423
521434229251664261223363
251423342122437262213423
253533233431734252213423
263333233431A46252213433
42343224A291366263221343
273335454432657253213423
2544323221126-6152213423
272645623234248252213423
452233444212619252212423
824434246141356262223313
251323233251524262213423
254533233332627232113223
232433442212337262213421
261423242122333262213423
5224341432219B7263223353
242313243252224252213423
524434246131366263223313
244313243262425252211423
142343242232325252213423
253533233432524232113223
241423442122433262213415
254655422137648253213423
522434144221897263123363
524435245141373463223343
244333342242333252213423
273645524234248253213423
251413542122338262212415
241313442122336252212415

24-locus MIRU-VNTR Patterns
273332444434749253213423
253533233433336252213423
254335444432658253213423
273335544434747253213423
271423442122234262213423
254313243252426252213523
233645326234247253213423
5224351442219A7263223353
273235444432648254213223
253533233433635212213423
522433171221BB82632233F3
223545524233248253213423
273235443432656254213423
273235344432658254213423
253533233433537232213223
5234352292714B4261222343
522434144221996262223363
6234332272414A4261223343
253535424244248253213423
424435246141346262223343
273335444432458253213422
251413442122232262212415
263235444432658254213423
221233243232335252213323
522434246141366264223313
264433433233235252213423
254313242252325252213423
252333144242233232213422
273545424434248253213423
255333233232325262213423
523436246171366263223313
5224341442219A7223223363
522426247231547262223363
273135444434247253213423
421435246141346263223342
243334444443648253213423
273545424224249253213423
271645224234247253213422
273335544432358255213423
5244332461413-6263223313
251333233252423261213423
255333243232425252213423
5234352292516C4261223333

24-locus MIRU-VNTR Patterns
463745343213447144223334
273335244432558253213423
252533233443235252213423
242343242232423252213423
293335443242458253213423
273335444443758253213423
5224341442218G7263223353
261423442122336262213423
262333243232322252213423
273335343434548253217423
233645324234248253213423
263335443442658253213423
273545824234248253213423
261233243231237252213412
243645424234248253213423
241324442122335252213415
273544524234243254113423
254313243252323252213323
221432142122247162213433
26374532-434249253213423
254313242250325252213423
253335444432648253213423
293335444432558253213423
5226341442219A6263223363
245432442232244252213423
5224341422219A7263223363
523435222261684261233353
273135444434545253213423
725434246141364263223313
163545324234248253213423
253533233433336252113423
254433442212447262213421
5224341442214B7263223353
283645424234248253213423
273545424233248353213423
261645424234237253213423
273635224232255253113423
244313343242425252213423
5224341442217A7263223343
27354552-232-41253213423
624534246141366263223313
253645324237248253213423
5224341442219A6263223343

24-locus MIRU-VNTR Patterns
152312242252322252213423
523425242221275261223343
253533233453637252113323
253746124234246253213424
525337346141363243223312
424434246141366263223313
5224331442218A7243223353
624434246141346263223313
251423442122437262213415
242322242232325252213423
524415246141386263223313
322434144221767263223363
233335243424248253213423
273336444432656253212423
5234352461412B6293223343
723415247241256353223343
273824445211845253213422
273334444434548253213423
262343242232325252213423
253533233433333252213423
254433533431546252213423
2733353-2432548253113423
923635247241964263224353
251333244212335252113423
151333243242325252211423
253533333433637252213432
5224341442219A2263223363
523435228251594261223343
253635224244248253213423
112513453433237252213423
252635523234248253213423
272645144234148253213423
273545324234246253213423
273644124234248253213423
25443232212223-152213423
215744424224435253213423
214734424224235253213424
271645424234244243213423
273336444432458253213423
27354552-234-45251213623
273545424235247253213423
212413346433248252203423
312435449241746263321343

24-locus MIRU-VNTR Patterns
263545424234248252213423
5223341442211A8263223383
5232352292718B4261223343
452233-4-212-14252112323
233233343212236252213413
421435247241866263224343
253645324234148253213423
252645424234247253213423
522433144221166263223353
214744324222245253213423
48223324-212-13252212423
273555422234246253213623
273724445211C48253213423
273335344433448252213433
513435247261B67273123353
34433344323251A252213423
29333444445263A253213423
273645423234247253213423
254213243232215252213423
272345724234257253213423
283454424224248253213423
233555424434145253213423
27354522-232-48253213423
A22434144221867263223363
2533322432-2535252113423
254433443433425252213423
27354442423424B253213423
273535524234248253213423
273355424235248253213423
323434239241765263223323
152333343262324152212432
251333243272322272213413
524435246161396264223423
253336341433549253213423
271645424234249253213623
5234352292416-3261223343
5224341442219F7263223363
36433334423241A252213423
273545224234248253213423
257532342122237162213423
5234363431413C42A3223353
312435249241B86263121343
272643523234248252213423

24-locus MIRU-VNTR Patterns
293235444432656254213423
263345524234248252213421
273545424233248253213423
233233243212337252213413
2-345452-234-48253213423
523336246141386263223313
252433342113237162213423
253232243221338252213413
5224341442217C7253223363
253633233433635252213423
253533133433534252113423
524634246141364263223313
5234352292515A4261223353
5226341442219A7263223363
112513444433237252213423
352333242232226252212423
251423322122237262213415
27383442-435-4-254214423
251333244212536252113423
253423223433536252213423
251423742124438262213415
251323343242425272213423
27333534-432-58253213423
255333242232525252113423
253223143232-35252213413
251423541122432262213315
254433433433438252213423
623425249221985261223323
273644324424247252213423
253645324254247253213623
212723142523238252213423
3223341442216A7262223363
34433344423241A252213423
224333242222324252213423
5224341442218B6263223353
622415246141376262223343
524334244141356264223313
253533233433437252213532
27333544444432A253213423
4224341442214A7264223363
523437226251774281223323
293435524234247253213423
623325247251236253223343

24-locus MIRU-VNTR Patterns
263135444432558253213423
352333343242319362213423
523325247271236253223343
522434144221587263223383
273435424234237253213422
523435244141365263223343
524435249221757261233343
522434144221957263223363
5224341442219C6263223363
272645424234248253213423
353333343232413252213423
522434144221886243223363
522434144221997222223363
5224341442219A8263323363
523434249281A75253223343
263334544432558253213423
271745424234244253213423
5234352461211E62A3223333
5224341442218A7163223373
323635229251775261223362
2A3235444432658253213423
2733354B3432557253212423
472335444432458253213423
263635424234267253213424
2B374442-434-4A153-13423
261223342122333262213423
273332434233348253213423
524425247241255253223343
254633433433437252213423
373335444432658253213423
273655324424248253213423
273645224234247253213423
523435322241655243223343
263334444432569263213423
523425247241226253223343
3543333442322 1A262213423
273645424254248253213423
623425246251255353213343
273335444433358253213422
283545524224237253213423
273645423234248253213423
5214341442219A7263223333
24332424443-748253213423

24-locus MIRU-VNTR Patterns
243433233433558252213423
253433253262334252213423
5234372272314A3261223343
273455424432258253213423
A22426225251256253223343
263336443534748253113423
252343242232425252213423
253423322212435262213421
254233433432438252213423
212513143433239252113423
274646424225248252213423
252334444432658253213423
722444144221777223223363
523535248241B86263123353
5224341442211B7263223353
5223341242218A8263223363
231412442132437262212415
523635228241774261223343
5224331442219A5263223353
72243524614135-263233343
252422342122232162213423
344333344232512252213423
272645324235248253213423
524434244141366273223313
241212442122237262212415
234333442212437262212421
523435229251684171223343
212513343433138352213423
522434144221896263223353
351413442122438262212415
5232352292713-4261223343
253233233232-37252213313
522434142221997263223373
254533433433337252213323
27333544343245A253213423
253232143232338252213412
243645324234247253213323
27354552-234647253213423
522434144221975263223363
152232233433535252213423
432233244212517252211423
522435229251644261223343
263332444432658253213423

24-locus MIRU-VNTR Patterns
3426442--443832242213323
263755422434244213113423
273335444444748252213423
073235444432568253213423
5224341442216A6263223353
254533133433735252213423
2533232442A2335252113324
254332243234423252213423
622425248231256353223343
424433247141366262223313
273314453432658253213423
252232122232323252212523
263545521244248253213423
273335344432655253213423
251323142122234262213524
253433233433437232213223
522434144221967222223363
273634424234248253213423
6224241442218B4263223363
263335444442658253213423
263334243222425252113423
244313342242425252213423
243433243442349252213423
7224341442219A72622233-3
073335444432558253213423
253335444432556253213423
254333253252336252212423
5224341442219A7262223373
2235233-4433435252113423
253333244232635252113423
353333244212416252212423
522433144221967263223353
522434144221937263223363
252335444434849253213423
522434144221797263223343
252343242242225252213423
4224331442214A6263223353
253523233433637232213233
274334444432558254213423
723425246251256253213343
252433342123235162213423
5224341442212A7263223363
273335444332658253213423

24-locus MIRU-VNTR Patterns
273335444432648253313423
27333544-252658253213423
4234252282913C4261223343
423224248281255253223323
722434144221957263123363
254432322122236162213413
274335444432557253213423
273335444412639253213423
6s2243414422189726322336 3
5234352461413A62A3223363
342743444443657242213322
5224352282615B2261223343
243523233432327232113223
524434246151266263223313
5224341442218A7243223363
253433133333537252113423
243233243232254252213412
272655824244147253213423
212922244433237252213423
5224341442218D7263223333
252344232232325252213423
522435225121377273223343
273325244443728253113423
5124331442219A7263223363
283335444432453253213423
261433333423526252213432
254433433433238222213423
5224341442219A7263223323
42342524A231246253223343
354333344232 51C252213423
255332321122235162213423
212313343433137252213423
5224341442216A7263223343
272645923234248253213423
233533433433325252213423
523436246151366263223313
522434144221747263223363
524434246141166264223313
353333243212414244212423
253523233433536252213423
283325444431748253213423
28355542443424422 3-13423
5224331442217A7263223363

24-locus MIRU-VNTR Patterns
273545424232245253213423
253535424234248253213423
5223231442219A7263223353
26354542423324C253213423
324336246141363263223313
322434144221867263223343
263645423434248253211423
2-3544524234295253213423
261432343423428252113432
624425246241256353223343
271643324234247253213423
28374452-434-4B253213423
273545-24234248253213323
-22434144221867263222363
254313243232425252213423
5224341442217A7263223373
283745424434248253213423
272645224234148253213423
424433247161365262223313
422334134221997262223363
273335344432657253213423
5224341442216C7263223353
3224341442213C7262223363
5224341442217B4273223363
522434144221827263223363
273335544432758253213423
5224341442217B7263223353
254323243232424252213423
522434144221955263223363
5224241422218A7263223363
523425244261265253223343
274345444432658253213423
5224331442217A7262223363
235313243262224252213423
522434144221887262223343
273533233433335252213423
253533233433537231213223
524434236141355263223313
275545124233248253213423
522434144221997263223373
283335344432656253213423
243644423234248253213423
523424246241251253223343

24-locus MIRU-VNTR Patterns
252335344433659253213423
273135444434648-53213423
5224331442217-7263223343
522434144221975262223363
253233243232438252213413
272335444432559253213423
246333233431346253213422
253645124234247253213423
251313442122236282212415
723436246151366263223313
253233143232218252213313
253533233433334252213423
2533232432C2438261113423
243223142232297262213411
253335444432557253213423
2s52233243232238252213413
523425246241251253223343
273335444434549253213423
524434245141363263223313
5236352292512B3261223353
5224331342218A7243223353
5224341242217A7263223384
523325247231256253223343
5224341442219A8263223343
422435245141367263223344
523425247241246243223343
273545423233249251213423
273335244432588253213423
273335444432476253213323
263834233473256252213422
2514234B2122338262213423
283565324234248253213423
233645424234148253213423
233413442212427262213421
265313243252325252213423
522434144221--4263223363
253233232232325262213423
253534233433735252213423
8224341442217A6262223363
223445129241966263223343
522434144221231263223363
273335243434648253213423
212513343433138252113423

24-locus MIRU-VNTR Patterns
252343242232324252213422
7s22434144221996262223363
272335444444848243213423
273545424234246253213423
283644424444247252213423
522434144221997263223323
273235474432258254213423
34274425-543-32242213323
6224341442211A8263223363
342333343232414252213423
374133344232514252213423
5224341442218A7263223323
264333242232225252213423
524535246141266263223313
522426247221546262223363
273235444434648251213423
273335444432258253213523
623425247251251353223343
274745224234247253213423
243223243232257252213311
251433433453338252213423
312435238241B86263-21343
251232133222223262203423
244433433433238252213423
254333243232325252213423
271335444432558253211323
822434144121995263223363
524434236141366263223313
523425246141366263223343
273335444432358253213423
27444552423424A253113423
233333254232237252213423
283734524234226253213423
5234352292713A4261223343
253433233423536252213523
273335454432558253213423
253325444432658253211423
251423541122438262113315
523334229251864261223343
25653233345354-152114423
524434246141365263222313
274644424444247222213423
5224341432219A7262223383

24-locus MIRU-VNTR Patterns
253323243232325262213423
243233243232333252213413
5221341442219A6263223363
1233352492416662B3221343
523425247241266253223323
352234444212417252212423
243233242232336252213213
273545724234248253213423
293545224234248253213433
253533133432327232113223
23341344221233726-213421
273335443232558253213423
252313243232325252213423
254333243232325262213423
273545424234248253214423
252353242232425252213423
273546524234238253213423
35433334322251B252213423
273445524234248252213423
524435246141366283223312
523635229241774261223343
355433433433335252213423
263335543432557253113423
255533233333436232213223
423635229251674241223343
25553243345323C262214423
23374542423424825З-13423
5234341442218A6263223363
252325154222337252213423
173545524234238253215423
423435237241764263221343
27343444-434644253213423
254313242252425252213423
261433242142238262213423
273633523232248253211423
5124341442219A7243223363
523425247241256254223343
283545424234248253214423
143734424234248253213423
423445247261664263221343
282645424234248253213423
273335544432458255213423
144343242222233252213423

24-locus MIRU-VNTR Patterns
253533233433734252213423
264235444442658254213423
233334344494648252213423
273235442432658251213423
5224441442219D7263223363
27333544443454725311 34-3
273335244432658233213423
256532333453562152114423
273325444464648253213423
155433223443349152213423
253233243242237252213413
254213243262425252213423
241233242232-37252213412
522434144221187262223363
253223243242257252213411
253533233423533252213424
263335344422558253213423
263335443432688253213423
251333243242425261213423
253433233433637232213223
252323243262525252213423
25364542-234-47253213423
241335444432358253213423
253313253232325252213423
273544524244248253213423
623325247241256153223343
524435247251366263123352
263233243232246232213412
27363532423422C253213423
473335444432558254213423
243546424223248253213423
35433334323251525-213423
257332342122215152213423
253423232433436252213423
251423442122337262213423
254433233454439252213423
253433433433335252213423
253223242232238252213413
524434246151356262223413
253223243232238252213413
4224341442218A7263223363
522435245141377273223342
271335434432458253211423

24-locus MIRU-VNTR Patterns
254213243252325252213423
2533332442A2435252113423
522435245141377273223343
273235444442658254213423
253533133433337152113423
252233242232258252213413
253533233433336152213423
273335444234747254213422
524436245141365262223314
5236352292A1772261223353
263455424434243283213423
141333243222233252213423
255333243232523242113423
273644524234243253113423
373645424234248253213423
5224341442217A5263223363
273545524234148152213423
153845324234248253113423
34274325-643-32242213323
263335424432654253213423
5224331442218A7263223363
273545521234647253213423
5224341442216B7253323353
34294425-453-32242213323
253643324234237253213223
251533233433437232113223
244432322122236162213423
26323544443265C253213423
233655424234248253213424
253533233443534252214423
5244352461313A6263223333
323425247241256353223343
522434144221AA7263223343
254433433443338252213323
255313122242424252213423
32243624A241866263221343
273644424444248252213423
273545224231268253213423
253335444430558252213423
273335434432558253213423
523435146141232273223343
243734424234248253213423
253233243232424252213413

24-locus MIRU-VNTR Patterns
272645424224248253213523
212513143533137252213423
524434245141266263223313
274644424444237252213423
253336343233349214213433
623325247241256253223363
523435229251684281223342
5226341442218A7263223363
253433243443449252213423
442232342212518252212413
2733353434335-8253213423
523625247241254253223343
243844424234247253213423
263834233473237252213423
623427247241256153223343
273545524234249253213423
253644524234248253213423
245333242242432252213423
244312252252425252213423
5224341422219A6263223373
263644224444247252213423
5224341442214A7263223343
2732304-4432458254213423
823424257241356253223343
344644224234156253213423
274335444432658253213433
424435249241436233223343
253233243232347252213412
251313243252125252213423
273645423234248263213423
253645324235248253213423
272334344431559253213423
253533233433537252213522
2B3234444332558254213423
254233242232328252213413
1s224141442219A7262213363
263325444432658253213423
2833354444326A5253213423
253533232243734252213423
233234343232337232213413
623425247241255252223343
233645524234247253213423
522434144221AF5263213363

24-locus MIRU-VNTR Patterns
273335444632558253213423
273335442432688253213423
234313243252525252213223
35433334423241A252213423
263232233232257252213411
252533233433438252213423
243223244262535252113423
252531233433436252213423
263335342424648253213423
3s3132324325252526221343 3
253645324234247254213423
522434144221896263223363
273334445430848252213423
522434142221977263223383
523425244291266253123343
423435244141347263223343
256433342143236162213423
263335443432658253213423
3s5353443347534625121342 3
253523133433637232213223
25343314349343A252213423
522534144221AA7263223363
523437245161367263223313
252343243232525252214423
27133544-422-54263211423
233334244233248253213423
212513143433136242213423
254432243423446252213423
4221341442218A7261223363
522434144221795263223363
255432343122635162213423
273335344432456253213424
273335444432556253213423
523235229271374261223343
254532322122226152213424
253335444432749254213423
3s7333544443265825321342 3
273335444432389253213423
273335444432437253213423
273335444434448253213422
273645424434247253213423
273345524234245254213423
27382444521164A253213433

24-locus MIRU-VNTR Patterns
252333154261336232213422
273335543442458253213423
272345444432458253213423
253644324231248253213423
273235444442659254213423
2523232-2232525252213423
253645323234265253213423
522534144221994263223363
254332142232226252213423
5224341442218-7242223363
27333444-432-57252213423
251333243252522262213423
243433233433535252213423
253334253240237252113423
271335444532558253211423
244433143463349252113423
522434144221956263223363
273646424234248252213423
261545524234248253213423
273545524234242253213423
26163542-223-46253213623
534434246131366263223313
243335444432658263213423
5224341442317E7262223363
354333344242517252213423
293335144432638253213423
273335344432257253213423
273724445211848253213433
5224341442218D7263223363
253412342122234162213423
253635423234247254213423
264235444432358253213423
274645423236137253213423
523635228241574261223353
273245424234247253213423
523525246141376263223343
252253323343827242111223
253433233433236232213223
275644424444247252213423
273335444432558253213421
522434144221-A6262225363
272824445211748263213433
243433233433518252213423

24-locus MIRU-VNTR Patterns
273335454434548273213423
153213242232225252213423
253533233412627232113223
253233233433526252213423
253433233453437252113423
263135444434647253213423
212513342433246252213423
273335443462558253213423
283645524234248253213423
252343242252425252213423
293335444432658253213423
271325444432558253211423
273335424432658252213423
324134245141362273223313
271335444432478253213423
273545423442258253213523
264645424434248253213423
273545324234249282213423
523535229291784171223343
261423442122338262213417
273335444432646253213423
224434246141366264223313
243335444434648252214423
253533233433636252213423
273335344432568253213413
263645424434148253213423
213722353433247252213423
524437246161365263223313
271335344433648253213421
523635228271374261223343
253234242242257252213412
8224341442217A7263223343
258422342122217162213423
274644424444248252213423
273545424234244253213423
273335441432658253213423
723425247241258253223342
253655324234245253213423
243665422433249213213423
423445248231A66283223343
243533233433536232113123
523435229231383281223343
272645824235246253213423

24-locus MIRU-VNTR Patterns
273135344434648252213423
244433233443537252213423
233533442212426262213421
253544324233248253213423
254232442122235162213423
253224243232332252113312
3s73823445211848253213423
623325247241256253223333
25553243343354B252214423
254314243282425252213423
6224272A6241256253223333
452233444212718252212423
273945424234237253213433
273235444432466254213423
212613343423128252213423
255523233433537252213323
241333243242525262213423
273445524232248252213423
273124434433648253213423
273335444432648253213423
421435249241935263121333
44223324421271A252212423
241323442120338242213415
163534233223326252113423
5234252472412A6253223323
234323242232525252213423
253633233433537252213423
282645823234248253213423
212712153433238252213423
252313242282325252213423
523424247241256253223344
271335443532648253211423
254433433233237252113423
273235444432655254213423
274334444433248253213423
173645224434148253213423
263233243242225262213423
5224341442219B7263223353
355333344222615252223423
623525247261256352223333
253523333433335252213423
251533233463337232113223
34433346423251A252213423

24-locus MIRU-VNTR Patterns
253533233434235252213423
273235444452658253213423
324416246141362263131313
253533233433535252213423
B23425247241254353223343
212A22243433238252213423
251233243232258252213412
522435249251225273223343
251423442122338262213423
5224341442218A7263213363
273824545211B38254213433
273545524224248242213423
423432249261526262221343
273334444432558253213423
262334444432657253213423
2512331B3232237252213413
275645423234238253213423
424434246141365272223313
263336443434746253213423
273335344432458252213424
173235244432559253213423
253665424433245253214423
251423142122438262212423
273124523234269253213423
6234352461413562B3223343
