# Supplementary material for: Universal genotyping reveals province-level differences in the molecular epidemiology of tuberculosis
Source: PLoS One. 2019 Apr 3;14(4):e0214870. doi: 10.1371/journal.pone.0214870 (PMC6447219; doi:10.1371/journal.pone.0214870)
Supplement: S3 Table — (PDF) [file pone.0214870.s003.pdf]

S3 Table. Multivariable analysis of factors associated with single and multi (≥2 isolates) contributors to an interprovincial 24-MIRU-VNTR cluster, Ontario and British Columbia 2008–2014.

Characteristic	Multi vs. Single **OR (95%CI)**	Multi vs. Single **aOR* (95%CI)**
Age, years		
0-14	1.4 (0.3–6.3)	1.0 (0.2–4.8)
15-34	Reference	Reference
35-54	1.5 (1.0–2.3)	1.4 (0.9–2.2)
55–74	1.2 (0.8–1.8)	1.2 (0.8–1.9)
75+	1.0 (0.6–1.7)	1.3 (0.7–2.1)
Sex		
Female	Reference	Reference
Male	1.3 (0.9–1.7)	1.2 (0.8–1.6)
Province of residence		
Ontario	Reference	Reference
British Columbia	0.6 (0.5–0.9)	0.6 (0.4–0.8)
Community type		
Metro	1.9 (1.0–3.8)	2.7 (1.2–5.8)
Urban, high-density	1.2 (0.6–2.4)	1.9 (0.9–4.1)
Urban, moderate-density	1.7 (0.7–4.0)	2.2 (0.9–5.7)
Rural/Remote	Reference	Reference
Birthplace		
Canada	3.3 (1.9–6.0)	8.0 (3.8–16.6)
Outside Canada	Reference	Reference
Lineage		
L1	1.0 (0.6–1.6)	2.4 (1.4–4.2)
L2	0.9 (0.6–1.4)	2.0 (1.2–3.5)
L3	0.6 (0.4–1.0)	1.4 (0.8–2.6)
L4	Reference	Reference

Abbreviations: OR—odds ratio; CI—confidence interval; aOR—adjusted odds ratio.
**Adjusted for age, sex, province, community type, birthplace, lineage.*
