# Supplementary material for: Universal genotyping reveals province-level differences in the molecular epidemiology of tuberculosis
Source: PLoS One. 2019 Apr 3;14(4):e0214870. doi: 10.1371/journal.pone.0214870 (PMC6447219; doi:10.1371/journal.pone.0214870)
Supplement: S1 Fig — (PDF) [file pone.0214870.s004.pdf]

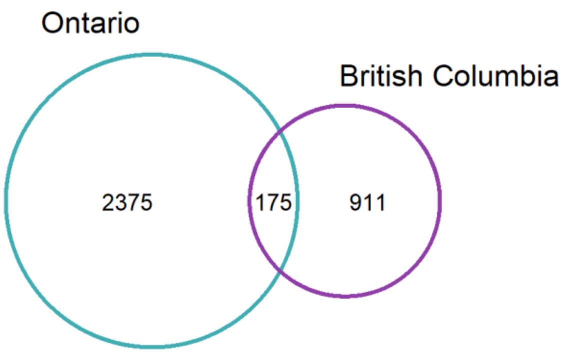

S1 Fig. Venn diagram representing the number of unique and shared 24-locus MIRU-VNTR genotypes between Ontario and British Columbia, 2008–2014.
