# Supplementary material for: Universal genotyping reveals province-level differences in the molecular epidemiology of tuberculosis
Source: PLoS One. 2019 Apr 3;14(4):e0214870. doi: 10.1371/journal.pone.0214870 (PMC6447219; doi:10.1371/journal.pone.0214870)
Supplement: S2 Fig — (PDF) [file pone.0214870.s005.pdf]

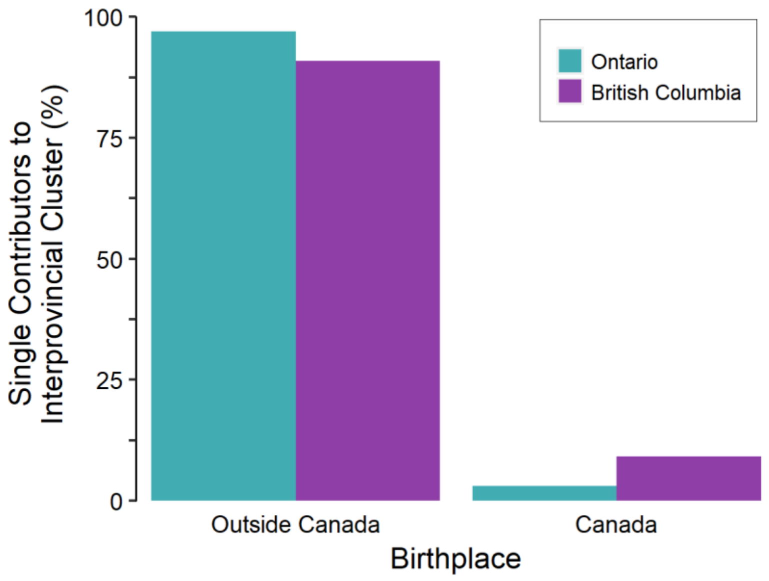

S2 Fig. Proportion of single contributors to an interprovincial cluster by province and birthplace, 2008–2014.
