# Supplementary material for: Universal genotyping reveals province-level differences in the molecular epidemiology of tuberculosis
Source: PLoS One. 2019 Apr 3;14(4):e0214870. doi: 10.1371/journal.pone.0214870 (PMC6447219; doi:10.1371/journal.pone.0214870)
Supplement: S3 Fig — Pies are scaled to the total number of isolates represented by each sub-lineage. (PDF) [file pone.0214870.s006.pdf]

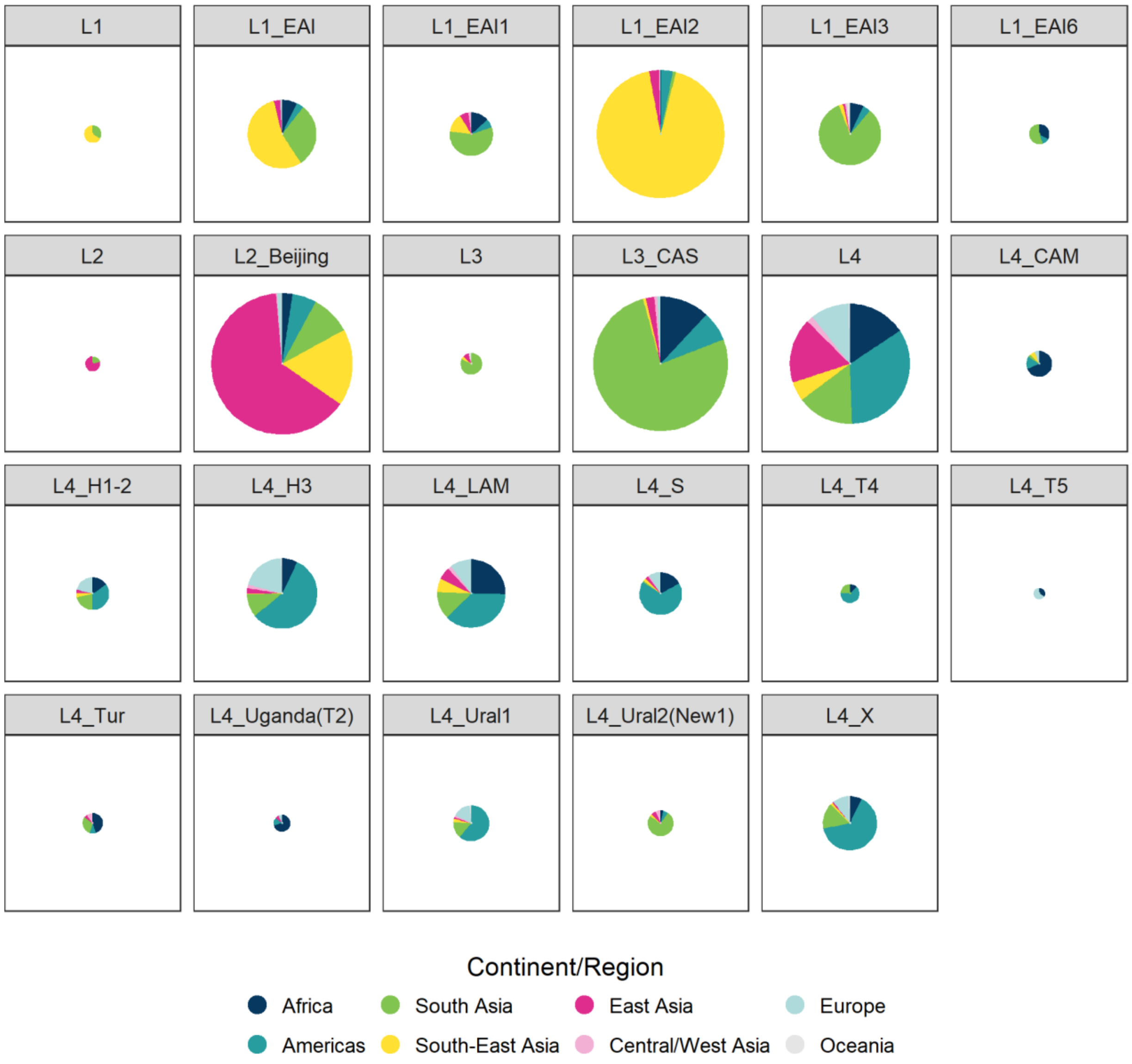

S3 Fig. Distribution of *Mycobacterium tuberculosis* sub-lineages in Ontario and British Columbia (2008–2014) by patient continent or region of birth. Pies are scaled to the total number of isolates represented by each sub-lineage.
